# Diagnostic efficacy of fecal-based miR-92a for advanced colorectal neoplasia: a prospective multicenter screening trial

**DOI:** 10.1186/s40779-025-00613-3

**Published:** 2025-06-13

**Authors:** Jia-Chen Wang, Li Zhao, Xiang-Yang Yu, Ting-Ping Wu, Chang-Fa Xia, Ju-Fang Shi, Hui He, Zhi-Qi Chen, Dan Shi, Han Xue, Qi Ao, Shu-Ping Liao, Zhang-Qiang Zheng, Qiong-Fang Huang, Lin Li, Sui-Ling Lin, Ying-Xue Li, Wen-Long Hu, Ji Peng, Lin Lei, Mao-Mao Cao, Fan Yang, Xin-Xin Yan, Si-Yi He, Meng-Di Cao, Shao-Li Zhang, Yi Teng, Qian-Ru Li, Nuo-Pei Tan, Hao-Yang Yu, Hong-Hui Cheng, Xi-Mo Wang, Wei-Qing Wu, Wan-Qing Chen

**Affiliations:** 1 Office of Cancer Screening, National Cancer Center/National Clinical Research Center for Cancer/Cancer Hospital, Chinese Academy of Medical Sciences and Peking Union Medical College, Beijing, 100021 China; 2 Department of Health Management, Shenzhen People’s Hospital, the Second Clinical Medical College of Jinan University/the First Affiliated Hospital of Southern University of Science and Technology, Shenzhen, 518053 Guangdong China; 3 Department of Gastrointestinal Surgery, Tianjin Nankai Hospital, Tianjin Medical University, Tianjin, 300000 China; 4 Department of Gastroenterology, Shenzhen Bao’an Traditional Chinese Medicine Hospital, Shenzhen, 518020 Guangdong China; 5 Department of Nursing, Tianjin Nankai Hospital, Tianjin Medical University, Tianjin, 300000 China; 6 Department of Gastroenterology, Shenzhen People’s Hospital, the Second Clinical Medical College of Jinan University/the First Affiliated Hospital of Southern University of Science and Technology, Shenzhen, 518053 Guangdong China; 7 Clinical Medical Research Center, Shenzhen People’s Hospital, the Second Clinical Medical College of Jinan University/the First Affiliated Hospital of Southern University of Science and Technology, Shenzhen, 518053 Guangdong China; 8 Shenzhen Center for Chronic Disease Control, Shenzhen, 518081 Guangdong China; 9 College of Life Sciences and Oceanography, Shenzhen University, Shenzhen, 518000 Guangdong China; 10 Tianjin Third Central Hospital, Tianjin Medical University, Tianjin, 300192 China; 11 Tianjin Third Central Hospital, Nankai University, Tianjin, 300192 China; 12 Tianjin Third Central Hospital, Tianjin University, Tianjin, 300192 China

**Keywords:** Advanced neoplasia, Colorectal cancer, Immunochemical fecal occult blood testing, MiR-92a, Screening

## Abstract

**Background:**

More efficacious, noninvasive screening methods are needed for advanced colorectal neoplasia. miR-92a is a reliable and reproducible biomarker for early colorectal cancer detection in stool samples. We compared the diagnostic efficacies of miR-92a, immunochemical fecal occult blood testing (FIT), and their combination (FIT + miR-92a) in a prospective multicenter screening trial.

**Methods:**

Overall, 16,240 participants aged 30–75 years were enrolled between April 1, 2021, and December 31, 2023. A total of 15,586 participants returned samples available for both FIT and miR-92a tests. All those with positive, and a random selection of those with negative screening tests were recommended to undergo colonoscopy. Follow-ups were performed until participants completed the colonoscopic examination. A total of 1401 screen-positive and 2079 randomly selected screen-negative individuals completed colonoscopies. Primary outcomes included sensitivity, number needed to screen (NNS), Youden index and receiver operating characteristic area under the curve (AUC) for advanced adenomas and colorectal cancer [advanced neoplasia (AN)] for each screening modality in the diagnostic performance analysis.

**Results:**

Colonoscopy was performed in 3480 individuals. The colonoscopy compliance rate was 47.8% for screen-positive individuals. The sensitivity of miR-92a versus FIT for AN was 70.9% versus 54.3% (*P* < 0.001), NNS was 24.7 versus 32.2 (*P* = 0.001), Youden index was 47.9% versus 35.0% (*P* < 0.001), AUC was 0.74 versus 0.67 (*P* = 0.010). FIT + miR-92a had a sensitivity of 85.4%, an NNS of 20.5, a Youden index of 47.9% and an AUC of 0.74 for AN.

**Conclusions:**

For AN screening, miR-92a demonstrated better sensitivity, NNS, Youden index and AUC as compared with FIT. Compared with FIT, using miR-92a appears to be more efficient for population-based screening programs. Screening sensitivity for AN can be further enhanced if conditionally used in combination with FIT.

***Trial registration*:**

Chinese Clinical Trial Registration Number: ChiCTR2200065415.

**Supplementary Information:**

The online version contains supplementary material available at 10.1186/s40779-025-00613-3.

## Background

Colorectal cancer (CRC) is the third most prevalent cancer and the second leading cause of cancer-related mortality globally [1]. In China, CRC morbidity and mortality have shown an increasing trend for decades [2], posing a significant public health challenge [3]. According to the latest data released by the National Cancer Center of China, in 2022, there were 517,100 new cases of CRC, representing 10.72% of all malignant tumors, and 240,000 deaths, accounting for 9.32% of all cancer-related deaths [4]. Notably, the number of new cases and age-specific rates of CRC have increased in young adults aged 20–49 years [5]. The majority of CRC cases evolve from precursor lesions known as colon adenomatous polyps or advanced adenomas (AA), of which a small fraction (3–5%) progress to cancer [6]. AA is considered an optimal target lesion for CRC prevention [7–9]. Early detection and intervention of AA can prevent the progression to CRC and reduce mortality [8–10]. According to the Surveillance, Epidemiology, and End Results (SEER) program, the 5-year survival rates of patients with early-stage and late-stage CRC were 90.9% and 15.1% from 2012 to 2018, respectively. In China, the proportion of late-stage CRC is 84.8%, while only 15.2% were detected in the early stage [11]. The stage at which the cancer diagnosis is made is crucial for determining patient survival rates, as early detection can greatly reduce mortality and prevent up to 90% of deaths [12]. Screening and early intervention are widely acknowledged as highly effective strategies to mitigate the incidence and mortality of CRC [13]. Colonoscopy, which is considered the gold standard for CRC screening, is an intricate and invasive diagnostic procedure [14]. A study conducted in 12 provinces of China revealed significant regional variations in colonoscopy compliance rates, with an overall screening compliance rate of only 15.3% [15]. The fecal occult blood test (FOBT) is a noninvasive screening option involving simple procedures and low cost. However, early screening with this approach is limited because of the absence of hemoglobin in the feces of patients with early CRC and AA [16]. A review has shown that the sensitivity of the fecal immunochemical test (FIT), an advanced version of FOBT at a 10 μg Hb/g cut-off for detecting advanced neoplasia (AN) is 0.27–0.40 [17]. The results of a comparative cross-sectional study of the performance of common FIT tests showed that the sensitivity for AN varied from 10.1 to 36.7%, and specificity varied from 85.5 to 96.6% [18]. According to the China guideline for the screening, early detection and early treatment of colorectal cancer (2020, Beijing), FIT is strongly recommended for CRC screening in China, with a high diagnostic sensitivity of 0.83 (95% CI 0.76–0.88) and specificity of 0.90 (95% CI 0.87–0.92) for CRC, but limited sensitivity of 0.36 (95% CI 0.28–0.45) with specificity of 0.92 (95% CI 0.89–0.94) for precancerous lesions [19]. The guideline states that FIT-positive individuals need to undergo colonoscopy for a definitive diagnosis [19]. Therefore, more sensitive and noninvasive screening techniques are urgently needed to enhance the detection of early CRC and AA, ultimately improving the 5-year survival rate of patients [20]. MicroRNAs (miRNAs) play crucial roles in tumor development and are potential diagnostic and prognostic biomarkers for cancer [21–23]. Evidence shows that miR-92a is a highly reliable marker for the early detection of CRC due to its inherent stability and reproducibility in stool samples [21, 23, 24]. By inhibiting the phosphatase and tensin homolog (PTEN) [25], the Krüppel-like factor 4 gene (KLF4) [26], and the Cyclin Dependent Kinase Inhibitor 1A (CDKN1A) [27], miR-92a promotes proliferation and migration of the tumor cells [24, 28]. A case-control study has demonstrated that miR-92a exhibited sensitivities of 71.6% (95% CI 70.0–80.7%) for the detection of early-stage CRC and 56.1% (95% CI 42.4–69.3%) for AA, with specificity of 73.3% (95% CI 63.5–81.6%) [24]. Consequently, miR-92a is a promising screening tool with high sensitivity and specificity for the early detection of CRC [24, 29]. However, the clinical utility of miR-92a, alone or in combination with the FIT, for large-scale population screening remains undetermined.

This large-scale prospective trial compared the diagnostic performance of miR-92a, FIT, and the combined FIT/miR-92a for AN screening, specifically assessing the diagnostic accuracy of miR-92a in a large screening population.

## Methods

### Study design and population

**Fig. 1:**
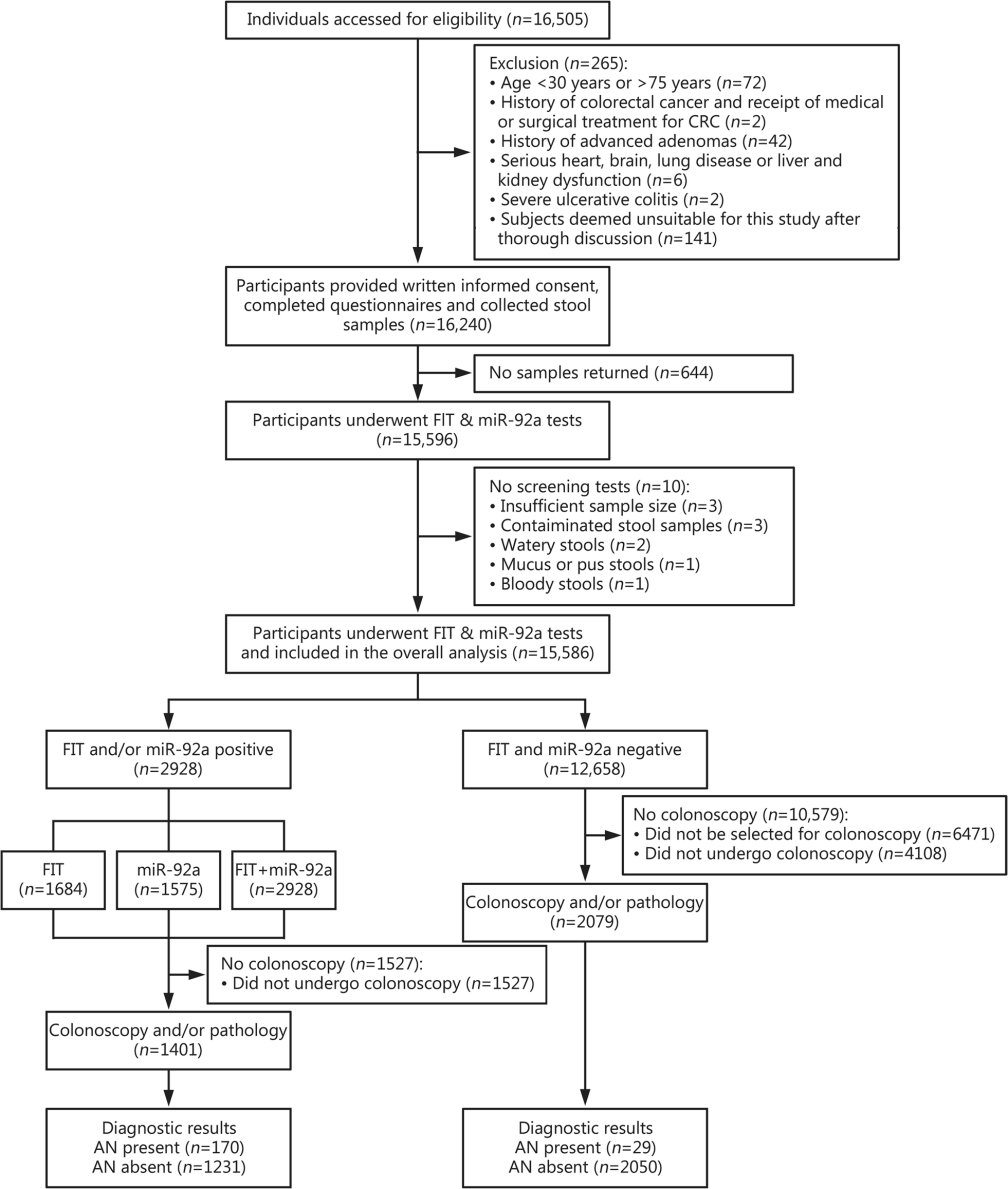
STARD 2015 flow diagram showing the flow of participants enrolled in the screening trial. FIT fecal immunochemical test, FIT + miR-92a immunochemical fecal occult blood test combined with miR-92a test, AN advanced neoplasia, CRC colorectal cancer

This multicenter prospective screening trial (ChiCTR2200065415), initiated in April 2021 enrolled participants from 3 clinical sites: Shenzhen People’s Hospital, Shenzhen Bao’an Traditional Chinese Medicine Hospital and Tianjin Nankai Hospital. Follow-ups were performed until participants completed the colonoscopic examination. A flowchart illustrating the process for inclusion and exclusion of study participants is presented in Fig. 1. We included individuals aged 30–75 years who could provide a qualified stool sample (Additional file 1) and written informed consent for colonoscopy. Exclusion criteria included prior diagnoses of CRC, AA, or polyps, as well as inflammatory bowel disease, hereditary CRC syndromes or withdrawal requests. Potential subjects were identified from the health examination department at each clinical center.

Ethical reviews were conducted separately by the ethics committees of each clinical center [Ethics review committee of Shenzhen People’s Hospital (SYL-202123-03); Ethics review committee of Shenzhen Bao’an District Hospital of Traditional Chinese Medicine (SJ-2021-001-04); Ethics review committee of Tianjin Nankai Hospital (NKYY_YX_IRB_2021_002)], with final approval from the group leader at Shenzhen People’s Hospital. Informed consent was obtained from all eligible participants.

### Procedures and data acquisitions

Trained healthcare staff assessed individuals from three clinical research centers (*n* = 16,505). After providing a thorough explanation of the screening trial process and obtaining informed consent, baseline questionnaires were administered to eligible participants, including questions on demographic information (age, sex, and smoking status), history of FOBT and colonoscopy, and family history of CRC in first-degree relatives. Data on a history of chronic diarrhea, chronic constipation, mucous stools, and other symptoms or signs of gastrointestinal disorders or psychological issues were collected. The baseline data were entered in a database by qualified personnel. In total, 15,596 eligible individuals underwent miR-92a and FIT simultaneously. The study protocol is shown in Additional file 1. Stool samples were collected at home and dropped off to the site staff on the same day. About 15% of the subjects were unable to return the samples on the same day and were asked to store the samples in ice packs. miR-92a test was completed within 4 weeks after the laboratory received the sample. Specific stool samples collection and storage instruction is shown in Additional file 1. Stool samples were examined by uniformly trained professionals following standardized protocols in each clinical center, and the outcomes were integrated logically by the specialized statistical analysts. All personnel involved in experimental work were blinded to the identities of samples. Stool samples were used for both FIT and miR-92a tests. Participants with positive miR-92a and/or FIT results were considered positive for FIT + miR-92a. Participants who tested positive were defined as individuals at high risk of AN and were recommended to undergo colonoscopy and/or pathology, which served as the gold standard for diagnosing AN (Additional file 2: Fig. S1). Individuals with negative screening results were randomly selected for colonoscopy, and the selection process of hierarchical randomization was shown in Additional file 1. Colonoscopy results were interpreted by skilled endoscopists in each clinical center.

### Screening and diagnostic protocols

#### Fecal miR-92a detection

The sample size required for fecal miR-92a testing is 0.3–0.5 g. To detect miR-92a levels in the feces, an miR-92a assay kit (REColon Nucleic Acid Extraction kit, Shenzhen GeneBioHealth Co., Ltd, Shenzhen, China) [30] was used to extract total RNA from stool samples using nucleic acid extraction reagents, followed by miR-92a specific reverse transcription-polymerase chain reaction (RT-PCR). A fluorescence quantitative PCR was employed to monitor the release of fluorescence signal in real-time, generating a curve to reflect the content of miR-92a in the sample. The predetermined positive criterion for miR-92a detection in an average-risk population was set at Cycle threshold (Ct) ≤ 32 by pre-experimentation prior to the clinical trial.

#### FIT

We used a qualitative FIT kit commonly used in China [Fecal Occult Blood Diagnostic Kit (Colloidal Gold), Qingdao Hantang Biotechnology Co., Ltd., Shandong, China] (Additional file 2: Table S1). The stool sample should be collected from various points (≥ 6 points) on the surface of the stool using a sampling rod. The collected sample should be mixed with the diluent-filled sample tube, ensuring thorough mixing by inverting it (Additional file 1). Subsequently, 2–3 drops of the mixed sample should be placed onto the test card and the “C” and “T” lines should be observed within 5 min. The presence of a red line at both the “C” and “T” lines indicated a positive result, while only a red line at the “C” line indicated a negative result. The absence of a red line at the “C” line indicated an operational error or reagent failure.

#### Colonoscopy and pathology

Subjects were instructed to orally consume 3000–4000 ml of polyethylene glycol in the evening before the examination. During the examination, the endoscopist adhered to standardized protocols to ensure a comprehensive visual examination. For patients with positive colonoscopy results, biopsies were performed, and all removed tissues were evaluated to determine lesion histology by gastrointestinal pathologists (Additional file 1). The cancer staging at diagnosis was assessed according to the American Joint Committee on Cancer staging manual 8 th edition [31], along with a detailed analysis of the number, location, size, and morphology of lesions. AA was characterized as an adenoma with a diameter ≥ 10 mm, and/or with a ≥ 25% villous component, and/or high-grade dysplasia [32]. Advanced neoplasia (AN) was defined as AA or CRC. If more than one lesion was discovered, the most advanced lesion was used to classify the participant.

### Outcomes

The outcomes of the analysis included positive rates (PR) and colonoscopy compliance rates. The compliance rate was defined as the percentage of individuals recommended for colonoscopy who completed the examination after a positive FIT and/or miR-92a. In the diagnostic performance analysis, primary outcomes were sensitivity, number needed to screen (NNS), diagnostic accuracy and diagnostic capacity. The receiver operating characteristics (ROC) analyses and the area under the curve (AUC) were used to evaluate the diagnostic accuracy. The Youden index was used to evaluate the diagnostic capacity of the screening method. The NNS was defined as the number of participants that had to be screened to detect 1 CRC or AA, or equivalently, the inverse of the true positive rate (TPR) [33]. The presence of AN was defined as a positive reference standard for these outcomes. Secondary outcomes included predictive value, likelihood ratio, and the detection rates (DR) of AN according to each screening modality. Modality-specific point estimates and confidence intervals (CIs) were provided for the outcomes. The definitions of the outcomes in this analysis are listed in Additional file 2: Table S2.

### Statistical analysis

Descriptive statistics were typically reported as the mean ± standard deviation (SD) for continuous variables, or as frequency counts and percentages for categorical variables. Paired results between FIT and miR-92a, including the PR, DR, sensitivity, specificity, and NNS were compared using McNamar’s test. The categorical variables were compared using the Chi-squared test or Fisher’s exact test when the expected count in one of the cells was below 5. The continuous variables were compared using Student’s *t-*test and Welch’s *t-*test for unequal variances. The positive predictive value (PPV) was compared using generalized estimating equation regression, and the corresponding *P-*value was reported based on the score test [34]. FIT + miR-92a was compared with miR-92a or FIT alone using McNamar’s test, followed with Bonferroni adjustment. The eXtreme Gradient Boosting (XGBoost) models were used separately to evaluate the characteristics affecting dropout in screen-positive and screen-negative individuals. A multivariate logistic regression model was used to examine how dropout affected the sensitivity and specificity of the screening tests (Additional file 2). The 95% confidence intervals (CIs) for NNS were also obtained indirectly, by inverting 95% confidence bounds for TPR, derived through normal approximation with binomial standard errors [33]. Significance was set at *P* < 0.05 (two-sided). All statistical analyses were performed using R version 4.2.3 software.

## Results

### Baseline characteristics

Between 2021 and 2023, we assessed 16,505 participants, excluding 919 based on predefined criteria (Fig. 1). Consequently, 15,586 participants were included in the final analysis (Fig. 1). Eligible participants completed informed consent forms and questionnaires (Additional file 2: Table S3). The baseline demographic characteristics and associated risk factors of all the participants are summarized in Table 1. Among all participants, 7072 (45.4%) were men, with an average age of (51.6 ± 7.0) years. No adverse events occurred during stool sample collection. Among 15,586 participants, 3480 (22.3%) participants underwent colonoscopies and were included in the diagnostic performance analysis to compare the diagnostic effectiveness of miR-92a, FIT, and FIT + miR-92a evaluations (Fig. 1; Table 2). The demographic characteristics and distribution of risk factors were summarized in Additional file 2: Tables S4–S7. Among them, 1461 (42.0%) were men, with an age of (53.9 ± 10.8) years. Age, sex, smoking history, family history of CRC, history of FOBT and colonoscopy, and symptoms or signs of gastrointestinal disorders were different significantly (*P* < 0.05) between participants who dropped out and those who fulfilled the study requirements (Additional file 2: Tables S4 and S5). The factors responsible for the high dropout rate among screen-positive and screen-negative individuals are illustrated in Additional file 2: Fig. S2. Age was the primary factor influencing dropout both in screen-positive and screen-negative individuals (Additional file 2: Fig. S2). A multivariate logistic regression model was employed on the expanded cohort (*n* = 15,586) to assess the effect of dropout on the sensitivity and specificity of the FIT, miR-92a, and FIT + miR-92a (Additional file 2: Table S8).

**Table 1:** Characteristics of participants included in the study (*n* = 15,586)

Characteristic	Value
Age (mean ± SD, year)	51.6 ± 7.0
Sex [*n* (%)]	
Male	7072 (45.4)
Female	8514 (54.6)
Cigarette smoking [*n* (%)]	
Yes	1644 (10.6)
No	13,907 (89.2)
Unknown	35 (0.2)
Cancer history other than CRC [*n* (%)]	
Yes	55 (0.4)
No	15,531 (99.6)
Family history of CRC^a^ [*n* (%)]	
Yes	155 (1.0)
No	15,431 (99.0)
History of FOBT within 5 years [*n* (%)]	
Yes (positive result)	97 (0.6)
Yes (negative result)	23 (0.1)
No	15,148 (97.2)
Unknown	318 (2.1)
History of colonoscopy within 5 years [*n* (%)]	
Yes	488 (3.1)
No	15,098 (96.9)
Symptoms or signs of gastrointestinal disorders [*n* (%)]	
Yes	1774 (11.4)
No	13,812 (88.6)
Symptoms or signs of psychological issues [*n* (%)]	
Yes	377 (2.4)
No	15,209 (97.6)

^a^First-degree relative (parent, sibling, child). *CRC* colorectal cancer, *FOBT* fecal occult blood test, *SD* standard deviation

**Table 2:** Positive rates between different groups

Characteristic	Total number (*n*)	Screening-positive participants [*n* (%)]			*P-*value
		FIT (*n* = 1684)	miR-92a (*n* = 1575)	FIT + miR-92a (*n* = 2928)	
Sex					
Male	7072	825 (11.7)	681 (9.6)	1339 (18.9)	< 0.001
Female	8514	859 (10.1)	894 (10.5)	1589 (18.7)	< 0.001
Age (years)					
30–35	1797	145 (8.1)	138 (7.7)	264 (14.7)	< 0.001
36–40	1677	148 (8.8)	133 (7.9)	257 (15.3)	< 0.001
41–45	1559	132 (8.5)	143 (9.2)	247 (15.8)	< 0.001
46–50	1989	163 (8.2)	186 (9.4)	319 (16.0)	< 0.001
51–55	2055	197 (9.6)	216 (10.5)	376 (18.3)	< 0.001
56–60	2231	236 (10.6)	251 (11.3)	429 (19.2)	< 0.001
61–65	2085	303 (14.5)	240 (11.5)	481 (23.1)	< 0.001
66–70	1601	258 (16.1)	201 (12.6)	408 (25.5)	< 0.001
71–75	592	102 (17.2)	67 (11.3)	147 (24.8)	< 0.001
Cigarette smoking					
Yes	1644	211 (12.8)	190 (11.6)	355 (21.6)	< 0.001
No	13,907	1470 (10.6)	1383 (9.9)	2569 (18.5)	< 0.001
Unknown	35	3 (8.6)	2 (5.7)	4 (11.4)	0.002
Cancer history other than CRC					
Yes	55	8 (14.5)	8 (14.5)	12 (21.8)	0.003
No	15,531	1676 (10.8)	1567 (10.1)	2916 (18.8)	< 0.001
Family history of CRC^a^					
Yes	155	16 (10.3)	21 (13.5)	31 (20.0)	< 0.001
No	15,431	1668 (10.8)	1554 (10.1)	2897 (18.8)	< 0.001
History of FOBT within 5 years					
Yes (positive result)	97	20 (20.6)	36 (37.1)	45 (46.4)	0.014
Yes (negative result)	23	5 (21.7)	8 (34.8)	11 (47.8)	0.363
No	15,148	1619 (10.7)	1492 (9.8)	2802 (18.5)	< 0.001
Unknown	318	40 (12.6)	39 (12.3)	70 (22.0)	< 0.001
History of colonoscopy within 5 years					
Yes	488	43 (8.8)	48 (9.8)	81 (16.6)	< 0.001
No	15,098	1641 (10.9)	1527 (10.1)	2847 (18.9)	< 0.001
Symptoms or signs of gastrointestinal disorders					
Yes	1774	218 (12.3)	206 (11.6)	383 (21.6)	< 0.001
No	13,812	1466 (10.6)	1369 (9.9)	2545 (18.4)	< 0.001
Symptoms or signs of psychological issues					
Yes	377	35 (9.3)	43 (11.4)	68 (18.0)	< 0.001
No	15,209	1649 (10.8)	1532 (10.1)	2860 (18.8)	< 0.001
Overall	15,586	1684 (10.8)	1575 (10.1)	2928 (18.8)	< 0.001

^a^First-degree relative (parent, sibling, child). *FIT* immunochemical fecal occult blood test, *FIT* + *miR-92a* immunochemical fecal occult blood test combined with miR-92a test, *CRC* colorectal cancer, *FOBT* fecal occult blood test

### Positive rate and colonoscopy compliance rate

The overall PRs were 10.1% (1575/15,586) for miR-92a, 10.8% (1684/15,586) for FIT, and 18.8% (2928/15,586) for FIT + miR-92a (Table 2). The PRs of FIT, miR-92a and FIT + miR-92a were significantly different among various subgroups (*P* < 0.05; Table 2). Additional risk-stratified subgroup analysis revealed that the PR of miR-92a was 10.5% in women and 9.6% in men (*P* = 0.08; Additional file 2: Table S9). PRs of miR-92a were elevated in individuals with risk factors, such as age > 50, history of FOBT, and symptoms or signs of gastrointestinal disorders, as compared with those without these risk factors (Additional file 2: Table S9).

A total of 2928 participants tested positive for miR-92a and/or FIT, of whom 1401 completed colonoscopies, resulting in a compliance rate of 47.8%. Stratified analysis showed significant differences in colonoscopy compliance rates among those considered at high risk based on demographics and other risk factors. Individuals with risk factors such as advanced age, family history of CRC, history of FOBT within 5 years, and symptoms or signs of gastrointestinal disorders exhibited higher colonoscopy compliance rates (*P* < 0.05; Additional file 2: Table S10).

### Primary outcomes

**Fig. 2:**
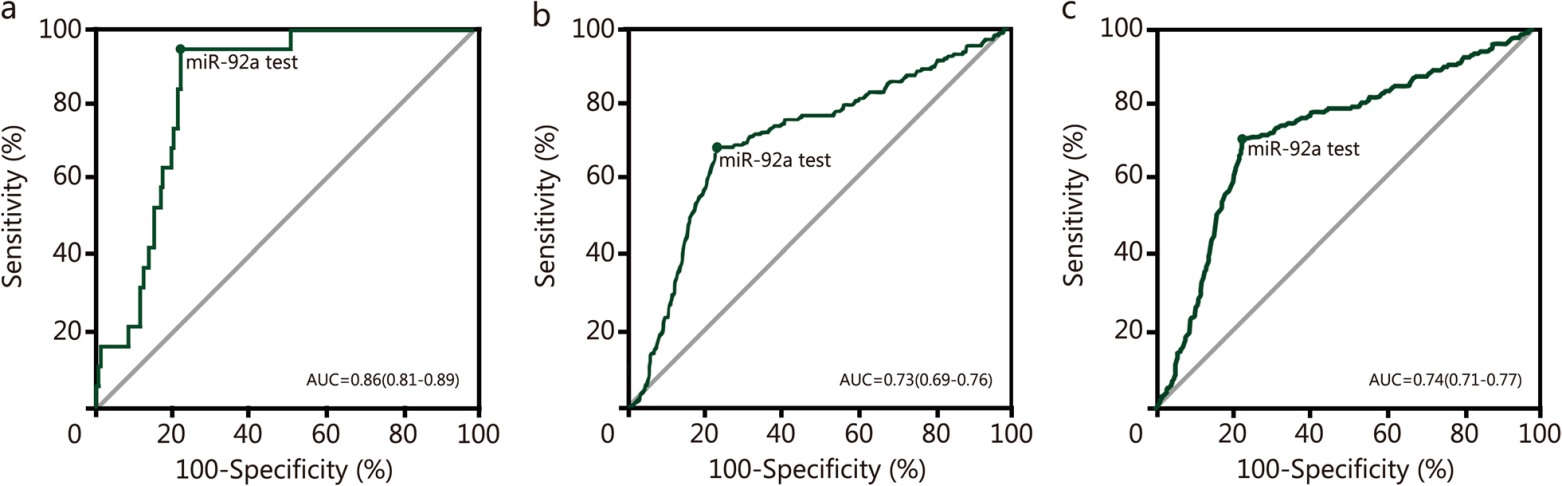
Sensitivity and specificity of miR-92a, FIT, and FIT + miR-92a for index lesions observed on colonoscopy. The miR-92a test receiver operating characteristic AUC evaluated CRC (**a**), AA (**b**) and AN (**c**) findings compared with all other findings (medium-risk adenomas, low-risk adenomas, and no findings). AA advanced adenoma, AN advanced neoplasia, AUC area under the curve, CRC colorectal cancer, FIT fecal immunochemical test, FIT + miR-92a immunochemical fecal occult blood test combined with miR-92a test

Based on the reference standard information, 199 participants had AN (19 CRC and 180 AA), while 3281 were cancer-free (Additional file 2: Table S11). The sensitivities of FIT and miR-92a were 84.2% (95% CI 60.4–96.6%) and 94.7% (95% CI 74.0–99.9%) for CRC screening, respectively (*P* < 0.001). The sensitivities of FIT and miR-92a were 51.1% (95% CI 43.6–58.6%) and 68.3% (95% CI 61.0–75.1%) for AA screening, respectively (*P* < 0.001). The sensitivities of FIT and miR-92a were 54.3% (95% CI 47.8–61.3%) and 70.9% (95% CI 64.0–77.1%) for AN screening, respectively (*P* < 0.001). For FIT + miR-92a, the sensitivity for screening CRC was 100.0% (95% CI 82.4–100.0%), 83.9% (95% CI 77.7–88.9%) for AA, and 85.4% (95% CI 79.7–90.0%) for AN. FIT + miR-92a significantly enhanced the screening sensitivity for AA and AN compared with miR-92a alone (*P* = 0.001). However, there was no significant difference in sensitivity between FIT + miR-92a and miR-92a in screening for CRC (*P* = 0.998). The specificities were 80.7% (95% CI 79.3–82.0%) for FIT, 77.0% (95% CI 75.5–78.4%) for miR-92a, and 62.5% (95% CI 60.8–64.1%) for FIT + miR-92a (*P* < 0.001; Table 3). The sensitivity for CRC was significantly higher by FIT, miR-92a and FIT + miR-92a than that for AA in almost all age groups (Additional file 2: Table S12). Additional risk-stratification subgroup analyses of sensitivity and specificity for AN were shown in Additional file 2: Tables S13 and S14. Sensitivity for AN was 61.3% versus 43.8% for FIT, 72.3% versus 68.8% for miR-92a, and 89.9% versus 78.8% for FIT + miR-92a, with specificity for other negative findings was 80.3% versus 85.9% for FIT, 87.7% versus 87.8% for miR-92a, and 69.4% versus 75.7% for FIT + miR-92a in men and women (Additional file 2: Table S13). The sensitivity for detecting proximal AA and distal AA were shown in Additional file 2: Table S15 (50.0% and 45.3% for FIT, 75.0% and 67.9% for miR-92a, 87.5% and 83.0% for FIT + miR-92a). The sensitivity and specificity of FIT, miR-92a, and FIT + miR-92a for AN after exclusion of previous FOBT-positive individuals were shown in Additional file 2: Table S16. The results of the multivariate logistic regression modeling showed that in the extended cohort (*n* = 15,586), miR-92a screened AN with a sensitivity of 66.3% (95% CI 62.2–70.8%) and a specificity of 91.9% (95% CI 91.5–92.4%) (Additional file 2: Table S8). The Youden index for detecting AN was 47.9% (95% CI 41.0–49.3%) for miR-92a and 35.0% (95% CI 27.8–36.3%) for FIT (*P* < 0.001). For FIT + miR-92a, the Youden index was 47.9% (95% CI 42.2–49.6%), which was not significantly different from it for miR-92a (*P* = 0.058; Table 3). ROC curves were generated for CRC, AA and AN compared with all other findings (medium-risk adenomas, low-risk adenomas, and no findings) by miR-92a. AUC was 0.86 (95% CI 0.81–0.89) for CRC, 0.73 (95% CI 0.69–0.76) for AA and 0.74 (95% CI 0.71–0.77) for AN by miR-92a (Fig. 2). The AUC of miR-92a versus FIT for AN was 0.74 (95% CI 0.71–0.77) versus 0.67 (95% CI 0.64–0.71) (*P* = 0.01). The NNS was 24.7 (95% CI 21.2–29.4) for miR-92a versus 32.2 (95% CI 27.8–39.6) for FIT (*P* = 0.001) for screening AN. For CRC screening, the NNS was 193.3 (95% CI 132.3–358.5) for miR-92a versus 217.5 (95% CI 146.1–425.5) for FIT (*P* = 0.248). For AA screening, the NNS was 28.3 (95% CI 24.1–34.2) for miR-92a versus 37.8 (95% CI 31.5–47.4) for FIT (*P* = 0.001). The NNS of FIT + miR-92a for screening AA and AN was significantly higher than miR-92a (*P* < 0.001), and was no statistical difference in screening for CRC (*P* = 0.997; Table 3).

**Table 3:** Diagnostic effectiveness indicators of different screening strategies (*n* = 3480)

Indicators	Screening strategies [% (95% CI)]			*P*_1_-value	*P*_2_-value
	FIT	miR-92a	FIT + miR-92a		
Sensitivity for CRC^a^	84.2 (60.4–96.6)	94.7 (74.0–99.9)	100.0 (82.4–100.0)	< 0.001^*^	0.998
Sensitivity for AA^a^	51.1 (43.6–58.6)	68.3 (61.0–75.1)	83.9 (77.7–88.9)	< 0.001^*^	0.001^*^
Sensitivity for AN^a^	54.3 (47.8–61.3)	70.9 (64.0–77.1)	85.4 (79.7–90.0)	< 0.001^*^	0.001^*^
Specificity^a^	80.7 (79.3–82.0)	77.0 (75.5–78.4)	62.5 (60.8–64.1)	< 0.001^*^	< 0.001^*^
Youden index for AN^b^	35.0 (27.8–36.3)	47.9 (41.0–49.3)	47.9 (42.2–49.6)	< 0.001^*^	0.058
AUC for AN	0.7 (0.6–0.7)	0.7 (0.7–0.8)	0.7 (0.7–0.8)	0.010^*^	0.989
NNS for CRC	217.5 (146.1–425.5)	193.3 (132.3–358.5)	183.2 (126.5–332.1)	0.248	0.997
NNS for AA	37.8 (31.5–47.4)	28.3 (24.1–34.2)	23.0 (19.9–27.3)	0.001^*^	< 0.001^*^
NNS for AN	32.2 (27.8–39.6)	24.7 (21.2–29.4)	20.5 (17.9–24.0)	0.001^*^	< 0.001^*^
PPV for AN^a^	14.6 (12.2–17.3)	15.8 (13.5–18.3)	12.1 (10.4–13.8)	< 0.001^*^	0.016^*^
NPV for AN^a^	96.7 (95.9–97.3)	97.8 (97.1–98.3)	98.6 (98.1–99.1)	0.001^*^	0.043
PLR for AN^c^	2.8 (2.4–3.3)	3.1 (2.8–3.4)	2.3 (2.1–2.6)	< 0.001^*^	0.001^*^
NLR for AN^d^	0.6 (0.5–0.7)	0.4 (0.3–0.5)	0.2 (0.1–0.5)	< 0.001^*^	< 0.001^*^

^a^Sensitivity, Specificity, PPV and NPV are presented by *n* (%); ^b^Youden index = Sensitivity + Specificity – 1; ^c^PLR = True-Positive Rate/False-Positive Rate = Sensitivity/(1 – Specificity); ^d^NLR = False-Negative Rate/True-Negative Rate = (1 – Sensitivity)/Specificity; *P*_1_-value was used between FIT and miR-92a using the McNemar’s test. *P*_2_-value was used between miR-92a and FIT + miR-92a using the McNemar’s test. Alpha was adjusted using the Bonferroni correction (α = 0.0167). *P represents a statistical difference. *FIT* immunochemical fecal occult blood test, *FIT* + *miR-92a* miR-92a in combination with immunochemical fecal occult blood test, *CRC* colorectal cancer, *AA* advanced adenoma, *AN* advanced neoplasia, *PPV* positive predictive value, *NPV* negative-predictive value, *PLR* positive-likelihood ratio, *NLR* negative-likelihood ratio, *NNS* number needed to screen, *CI* confidence interval

### Secondary outcomes

The PPV was 15.8% (95% CI 13.5–18.3%) for miR-92a versus 14.6% (95% CI 12.2–17.3%) for FIT for screening AN (*P* < 0.001; Table 3; Additional file 2: Table S17). The positive-likelihood ratios (PLR) and the corresponding negative-likelihood ratios (NLR) for FIT, miR-92a, and FIT + miR-92a were shown in Table 3 and Additional file 2 Table S18. A total of 199 AN cases (19 CRC and 180 AA) were detected in 3480 colonoscopies (Table 3; Additional file 2: Table S11). miR-92a detected 141 AN cases (18 CRC and 123 AA), whereas FIT detected 108 AN cases (16 CRC and 92 AA). FIT + miR-92a detected 170 AN cases (19 CRC cases and 151 AA cases) (Additional file 2: Table S19). In 1401 cases of colonoscopy in the screen-positive population detected by FIT + miR-92a, the overall DR of AN was 12.1% (*n* = 170), including 19 CRCs and 151 AAs. The DR of AN by each screening modality were shown in Additional file 2: Table S19. CRC primarily affected the sigmoid colon (36.84%). The AA are often multiple (83.33%), with more than one lesion often involving the ascending, transverse, and sigmoid colons. The median size of AA was 1.0–2.0 cm (Additional file 2: Table S20).

## Discussion

To detect AN, FIT, the traditional stool-based screening method, achieved a sensitivity of 54.3%, whereas miR-92a and FIT + miR-92a exhibited higher sensitivity (70.9% and 85.4%, respectively). The Youden index of miR-92a was significantly higher than that of FIT. The NNS for AN by FIT + miR-92a was the lowest (20.5), whereas the NNS of miR-92a and FIT were 24.7 and 32.2, respectively. For AN screening, miR-92a demonstrated better sensitivity, NNS, Youden index and AUC as compared to FIT. Compared with FIT, using miR-92a appears to be more efficient for population-based screening programs. Screening sensitivity for AN can be further enhanced if conditionally used in combination with FIT. Our study provides evidence demonstrating that miR-92a is a potential biomarker for noninvasive screening of CRC and AA.

A previous Meta-analysis based on many case-control studies revealed that fecal-based miR-92a testing for CRC detection demonstrated a sensitivity of 46.8% (95% CI 26.3–68.4%) and specificity of 90.5% (95% CI 77.1–96.4%), while miR-92a testing for AA detection exhibited a sensitivity of 43.2% (95% CI 20.1–69.8%) and specificity of 91.0% (95% CI 41.6–99.3%) [35]. A previous study used real-time quantitative polymerase chain reaction (RT-qPCR) to detect miR-92a in stool samples and found a sensitivity of 71.6% (95% CI 70.0–80.7%) and a specificity of 73.3% (95% CI 63.5–81.6%) in 88 patients with CRC [24]. Compared to previous studies, our study improved the screening sensitivity of miR-92a for AN and especially for AA, which may be associated with the use of optimized miR-92a primers, probes, and key reagents (e.g., reverse transcriptase) in the miR-92a assay kit that we used. Our study is a large-scale prospective screening trial in China validating the effectiveness of miR-92a for early CRC detection in an average-risk population, and comparing its effectiveness with the combination of FIT. miR-92a is a promising marker for detecting AA than FIT, with higher sensitivity (70.9% versus 54.3%) and lower NNS (24.7 versus 32.2). When miR-92a and FIT were used in combination, there was a further increase in sensitivity, accompanied by a further decrease in NNS. Therefore, we recommend screening with miR-92a in combination with FIT in areas with abundant medical resources.

In this prospective screening trial, multiple risk factors were assessed. Stratification by sex showed that there was no significant difference between the positive rates of miR-92a in men and women. Stratification by age showed a consistent increase in screening participation with increasing age. This may be because the incidence of adenomas is higher in older individuals, and because of the education provided during the pre-questionnaire. In turn, older patients were more willing to undergo colonoscopy. Previous study has reported a two-fold higher detection rate for AN in men than in women based on colonoscopy [36], which highlights the need to consider the sensitivity of screening programs for women. The DR of miR-92a was 2.0% for CRC, and was 13.7% for AA in our study. A previous CRC screening trial, which also based on the average-risk population in China, showed a DR of 1.9% for CRC and 13.4% for AA by a blood-based multilocus DNA methylation assay [37]. The results of the systematic review showed that, compared with no screening, colonoscopy screening reduced the risk of CRC morbidity by 56% (*RR* = 0.44, 95% CI 0.22–0.88) and the risk of CRC mortality by 57% (*RR* = 0.43, 95% CI 0.35–0.53), while FIT screening reduced the risk of CRC mortality by 52% (*RR* = 0.48, 95% CI 0.20–1.12) [17]. Although colonoscopy and FIT have been used to enhance early diagnosis for various decades, CRC continues to have a high incidence and mortality in China [4, 38]. Considering the limitations of the current screening programs, such as a low compliance rate with colonoscopy and low sensitivity of FIT for AA [39], exploring new noninvasive screening tools is clearly crucial. Previous studies have reported a notable elevation in the expression of miR-92a in CRC and AA [35, 40]. Fecal-based miR-92a has also shown promise for clinical applications in CRC screening. Huang et al. [41] reported that miR-92a levels exhibited diagnostic value for CRC, with an impressive area under the curve of 83%. Our study showed that the AUC for AN was 0.74 by miR-92a. These results indicate the potential of miR-92a as a promising and effective screening tool for early detection of CRC and AA. Our prospective screening trial provides robust evidence supporting the effectiveness of miR-92a as a screening marker for AN, and highlights its potential for clinical application.

Colonoscopy was considered as the definitive diagnostic test in our study. However, in population-based screening programs in China, suboptimal adherence to colonoscopy has always been a prominent concern [42]. The compliance rate among individuals who tested positive by FIT and miR-92a in this study was 47.8% (1401/2928). CRC Screening Program in Urban China (canSPUC) [2] and Coloclear (stool DNA assay-based screening program) [43] showed a colonoscopy compliance rate of 14.0% and 29.4%, respectively. The most common reasons for refusing colonoscopy are “a lack of time”, “lack of significance”, and “fear of pain during invasive testing” [2]. XGBoost models were used separately to evaluate the characteristics affecting dropout in screen-positive and screen-negative individuals. The sensitivity and specificity of the predictive modeling show stability. The screening effectiveness of miR-92a, even with low colonoscopy adherence, shows promise in average-risk population screening. However, low compliance rates can lead to critical issues, including insufficient diagnostic follow-up (e.g., fewer participants undergoing colonoscopy), which may introduce substantial bias into the study results.

Our study highlights the importance of ongoing public outreach efforts to enhance CRC screening efficacy. We believe that, compared with FIT, using miR-92a for AN screening might be more efficient in population for various reasons. First, miR-92a demonstrated higher screening sensitivity compared with FIT, which can be further increased by using FIT + miR-92a. Considering the high malignancy of CRC, detection at an early stage, such as AA, is particularly important for the prevention of CRC. FIT + miR-92a and miR-92a have significantly improved sensitivity in screening for AA compared to FIT. Second, the NNS of miR-92a was significantly lower than that of FIT, which can save the medical resources to a great extent. Third, the AUC and Youden index of miR-92a, which took both sensitivity and specificity into account, were significantly higher than FIT. Fourth, the kit of FIT and miR-92a used was noninvasive, convenient, and inexpensive, so that people can do at-home sampling. The FIT kit costs approximately ¥ 15–20, while the miR-92a kit, which is covered by medical insurance, has a price of ¥ 400. It is undeniable that an increase in sensitivity is inevitably accompanied by a decrease in specificity. Although the specificity of miR-92a and FIT + miR-92a screening is lower than that of FIT, given the high malignancy of CRC and the purpose of the primary screening test, the sensitivity of the screening methods is of greater concern. In general, sensitivity should be given more weight than specificity either when the disease prevalence is higher or when the disease severity is greater. Our goal was to explore whether the miR-92a assay has the potential to be a means of primary screening for AN, and therefore more attention was paid to sensitivity. In this study, we adopted a parallel design (classifying a subject as positive if either test is positive) to maximize diagnostic sensitivity. A tandem design (classifying a subject as positive only if both tests are positive) was not considered, as it requires more healthcare resources and yields lower sensitivity, which does not align with our goal of providing practical guidance for diverse healthcare settings in China. Because of the higher sensitivity of miR-92a and FIT + miR-92a, it may be possible to decrease the frequency of screening, but this would have to be examined in future studies. Therefore, the use of miR-92a would be advantageous, and FIT + miR-92a would further increase screening sensitivity.

Our study had several notable advantages. First, no previous large-scale prospective screening trial based on an average-risk population in China has assessed the effectiveness of a novel noninvasive screening test, miR-92a. We also evaluated its combined use with FIT. Second, our results show that miR-92a testing is significantly more effective than FIT in screening for AA (precancerous stage) and can better achieve CRC prevention. Third, we collected extensive epidemiological survey questionnaires and clinical information from the participants. Data annotation was performed by experienced professional doctors and laboratory staff members through three-level quality control annotation to ensure that the dataset was of high annotation quality. However, this study also had some limitations. First, colonoscopy compliance rates both among screen-positive individuals and randomly selected screen-negative individuals were not very high, which is a common issue in real-world CRC screening for average-risk population. The study sample being weighted toward people more likely to have CRC and AA by virtue of more FIT, miR-92a, or FIT + miR-92a positive individuals undergoing colonoscopy, which may have overestimated the sensitivity of screening tests. At the same time, because some of the randomly selected screen-negative individuals did not complete a colonoscopy, it means that the samples of screen-negative individuals who underwent colonoscopy were not a true random sample. Second, miR-92a screening for AN was slightly less specific than was FIT screening, which we have already discussed it before.

## Conclusions

In summary, our study assessed the effectiveness of miR-92a in detecting CRC and its precursors in an average-risk population. For AN screening, miR-92a demonstrated better sensitivity, NNS, Youden index and AUC compared to FIT. Screening sensitivity for AN can be further enhanced if conditionally used in combination with FIT. The screening effect will be further enhanced when combined with FIT. Genetic testing is a novel screening approach that can improve the effectiveness of future screening modalities by complementing other tests. Our findings provide valuable insights into the development of effective population-based screening strategies for AN.

## Supplementary Information

**Additional file 1.** Trial protocol

**Additional file 2.** **Table S1** FIT Brands, Hemoglobin Detection Level and Stability and Storage Information. **Table S2** The definitions of outcomes in this study. **Table S3** Survey questions and responses provided by participants at the time of enrollment.** Table S4** Demographics and socioeconomic status for participants who were enrolled but did not complete all study requirements (withdrawn cohort) compared to those who completed all study requirements (eligible cohort).** Table S5** Symptoms or signs of gastrointestinal disorders or psychological issues for participants who were enrolled but did not complete all study requirements (withdrawn cohort) compared to those who completed all study requirements (eligible cohort) [*n* (%)].** Table S6** Demographic and socioeconomic risk factors and symptoms or signs of gastrointestinal disorders or psychological issues for screening-positive (FIT positive and/or miR-92a positive) participants who were enrolled but did not complete all study requirements (withdrawn cohort) compared to those who completed all study requirements (eligible cohort).** Table S7** Demographic and socioeconomic risk factors and symptoms or signs of gastrointestinal disorders or psychological issues for screening double-negative (FIT negative and miR-92a negative) participants who were enrolled but did not complete all study requirements (withdrawn cohort) compared to those who completed all study requirements (eligible cohort).** Table S8** Logistic regression model to assess the impact of dropout on the FIT, miR-92a and FIT + miR-92a sensitivity and specificity [% (95% CI)]. **Table S9** Positive rate by miR-92a test between different groups. **Table S10** Compliance rates among high-risk population between different groups. **Table S11** FIT, miR-92a and FIT + miR92a screening for AN.** Table S12** Sensitivity and specificity of the miR-92a and FIT + miR-92a test by age [% (95% CI)].** Table S13** Sensitivity and specificity by demographic and socioeconomic risk factors: sex, smoking, history of FOBT within 5 years, history of colonoscopy within 5 years and family history of CRC [% (95% CI)].** Table S14** Sensitivity and specificity by different symptoms or signs of gastrointestinal disorders or psychological issues for participants [% (95% CI)].** Table S15** Sensitivity of FIT, miR-92a and FIT + miR-92a for subcategories of colorectal cancer (CRC) (*n* = 19) and advanced adenomas (AA) (*n* = 180) [% (95% CI)].** Table S16** Sensitivity and specificity of different screening strategies for screening AN (*n* = 3415) [% (95% CI)].** Table S17** Positive predictive value (PPV) and negative predictive value (NPV) for FIT, miR-92a and FIT + miR-92a.** Table S18** Diagnostic likelihood ratios (DLRs) for FIT, miR-92a and FIT + miR-92a [% (95% CI)].** Table S19** Detection rates (DRs) of CRC, AA and AN by FIT, miR-92a and FIT + miR-92a (*n* = 3480) [% (95% CI)].** Table S20** Characteristics of advanced neoplasias [*n* (%)]. **Fig. S1** Flowchart for colonoscopy and/or pathology for different screening results. **Fig. S2** XGBoost classification models illustrated the influence of various factors contributing to dropout

## Data Availability

Due to current ethical approval for the study, individual participant’s complete data will not be shared. Descriptive data in table as well as data analysis methods are available upon request. Please contact corresponding author Professor Wan-Qing Chen at chenwq@cicams.ac.cn for access.

## Abbreviations

AA: Advanced adenomas
AN: Advanced neoplasia
AUC: Area under the curve
CDKN1A: Cyclin dependent kinase inhibitor 1A
CIs: Confidence intervals
CRC: Colorectal cancer
Ct: Cycle threshold
DR: Detection rate
FIT: Immunochemical fecal occult blood test
FOBT: Fecal occult blood test
KLF4: Krüppel-like factor 4 gene
NLR: Negative-likelihood ratio
NNS: Number needed to screen
NPV: Negative-predictive value
PLR: Positive-likelihood ratio
PPV: Positive-predictive value
PR: Positive rate
PTEN: Phosphatase and tensin homolog
ROC: Receiver operating characteristics
RR: Relative risk
RT-PCR: Reverse transcription-polymerase chain reaction
SD: Standard deviation
TPR: True positive rate
XGBoost: eXtreme Gradient Boosting
