# Supplementary material for: Diagnostic efficacy of fecal-based miR-92a for advanced colorectal neoplasia: a prospective multicenter screening trial
Source: Mil Med Res. 2025 Jun 13;12:30. doi: 10.1186/s40779-025-00613-3 (PMC12164163; doi:10.1186/s40779-025-00613-3)
Supplement: Supplementary file 1 — Additional file 1. Trial protocol [file 40779_2025_613_MOESM1_ESM.pdf]

Prospective study of the fecal-based miR-92a test for screening advanced colorectal neoplasia in a general risk population

Genebiohealth, Inc.	Program number: CASC01
	Version number: 202202
	Version date: 2022.2.27
	Registration authority: China Clinical Trial Registry (ChiCTR)
	Registration number: ChiCTR2200065415

Table of Contents

0. Abbreviations 1
1. Introduction of the test 1
    1.1 Title of the study 1
    1.2 Clinical institutions 1
    1.3 Aims of the trial 1
    1.4 Study population 2
    1.5 Project schedule 2
2. Project introduction 2
    2.1 Background 2
    2.2 Fecal miR-92a detection technology 3
    2.3 Research content 4
3. Research target 5
4. Research methods 5
    4.1 Colorectal cancer risk questionnaire interview 5
    4.2 Fecal-based miR-92a test 5
        4.2.1 Principle 5
        4.2.2 Main components 5
        4.2.3 Storage conditions and expiration dates 5
        4.2.4 Procedure 6
    4.3 Fecal occult blood test 6

4.3.1 Principle 6
4.3.2 Main components of the kit 7
4.3.3 Storage conditions and expiration dates 7
4.3.4 Procedure 7
4.4 Colonoscopy 8
4.5 Screening experiment design 8
5. Study population 9
5.1 Enrolment of subjects 9
5.2 Inclusion criteria 9
5.3 Exclusion criteria 9
5.4 Removal criteria 9
5.5 Sample size considerations 10
6. Research program design and processes 11
6.1 Subjects enrolled 11
6.2 Sample reception and storage 12
6.3 Fecal-based miR-92a test 12
6.4 Fecal occult blood test 12
6.5 Colonoscopy and/or pathology 12
6.6 Follow-up management 12
6.6.1 Subjects tested positive for fecal samples 13
6.6.2 Subjects tested negative for fecal samples 13
6.7 Information entry and collation 14
6.8 Research materials and equipment 15
6.9 Back-up sample storage 15
7. Data analysis 15
7.1 Test termination points 15
7.1.1 Relevant definitions 15
7.1.2 Categorization of participants based on colonoscopy and histopathology 16
7.1.3 Endpoint 17
7.2 Analysis of clinical outcomes 18
7.3 Other research statistics 19

7.4 Statistical methods 19
8. Ethical issues 19
8.1 Risk analysis 19
8.1.1 Subject risks 19
8.1.2 Information confidentiality 20
8.1.3 Subject benefits 20
8.1.4 Explanation of test safety 21
8.2 Informed consent 21
8.3 Data reliability 21
8.4 Project management 21
8.5 Ethics review committee 21
9. Instructions for stool sample collection 22
10. References 23

0. Abbreviations

AA	Advanced adenomas	NPV	Negative predictive value
AN	Advanced neoplasia	PCR	Polymerase chain reaction
CA	Carbohydrate antigen	PPV	Positive predictive value
cDNA	Complementary DNA	RNA	Ribonucleic acid
CEA	Carcinoembryonic antigen	RT-PCR	Reverse transcription polymerase chain reaction
CRC	Colorectal cancer	SN	Sensitivity
DR	Detection rate	SP	Specificity
GEE	Generalized estimating equation	TNM	Tumor node metastasis classification
Hb	Hemoglobin	TNR	True negative rate
Ig	Immunoglobulin	TPR	True positive rate
NEG	Negative		

1. Introduction of the test

1.1 Title of the study

Prospective study of the fecal-based miR-92a test for screening colorectal cancer in a general risk population.

1.2 Clinical institutions

Shenzhen People's Hospital (SZH) (head unit), Shenzhen Bao'an Traditional Chinese Medicine Hospital (BAT), and Tianjin Nankai Hospital (NKH).

1.3 Aims of the trial

The project is to perform fecal-based miR-92a test and immunochemical fecal occult blood test (FIT) on people at average risk of colorectal cancer (CRC). The performance of FIT and miR-92a and their combination for CRC and advanced adenomas (AA) [advanced neoplasia (AN)] will be analyzed by using colonoscopy and/or pathological findings as the gold standard, and the performance indicators including sensitivity, specificity, predictive values, diagnostic accuracy, likelihood ratios and overall compliance rate will be evaluated, to form an early screening and prevention strategy for the population at average risk of CRC.

1.4 Study population

This is a prospective study of a population at average risk for CRC. 16,505 subjects aged from 30 to 75 will be enrolled in this prospective study based on inclusion and exclusion criteria, if they are fully informed about the study and sign the informed consent. The investigators will continue to monitor the progress of enrolment and the rate of positivity for miR-92a, after the start of the study to determine whether enrolment should be terminated early.

1.5 Project schedule

- The clinical trial protocol was initially submitted for ethical review in April 2021, and the ethical review was completed by the leading unit in May 2021;
- The filing and approval of human genetic resources were completed in June 2021, followed by the completion of ethical review by the participating medical institutions in June 2021;
- Starting from July 2021, the screening and enrollment of subjects began, followed by information entry, collection of fecal samples, laboratory testing of fecal samples, and colonoscopy;
- Colonoscopies were undergone starting from November 2021, for subjects who were required to undergo the procedure according to the study protocol design;
- The data analysis and general report of the clinical trial were completed by the end of December 2023.

2. Project introduction

2.1 Background

CRC is one of the top-ranked cancers in terms of incidence and mortality both in China and globally. According to GloboCan2020, CRC accounts for 1,148,515 new cases and 576,858 deaths globally, ranking as the fifth in terms of global incidence among the most common cancers and holds the fifth highest mortality rate worldwide [1]. According to the relevant statistics in China, the incidence and death rate of CRC ranked 5th among all malignant tumors in 2017, with 376,000 new cases and 191,000 deaths [2]. Colonoscopy is currently the main diagnostic tool for CRC, but the complexity of the examination process, the low compliance of the test and the low accuracy of the preliminaries limit the advantages of colonoscopies. For example, if the patient's bowel is not adequately prepared or if the early lesions are not clearly visible on microscopy, these lesions are more likely to be missed. Furthermore, the substantial population at risk for CRC, particularly due to advancing age, coupled with the concentration of CRC epidemiology in economically

developed regions, presents challenges for colonoscopy to emerge as the primary screening method for individuals at risk [3]. This is attributed to issues such as low compliance rates and prolonged appointment waiting times, especially in regions with high incidence rates of the disease. In addition to colonoscopy, FIT is a commonly used primary screening tool for people at risk of CRC. The test is based on the qualitative detection of human hemoglobin in fecal samples by immunocolloidal gold assay, which is relatively simple and inexpensive to perform. Nevertheless, the limitations of the testing technology and result interpretation principle only allow for the detection of blood in fecal samples. Consequently, there may be a lack of effective correlation between the test results and the detection of pre-cancerous and high-risk lesions, especially adenomas in their advanced stages of development. While other blood-based markers such as carcinoembryonic antigen (CEA) and carbohydrate antigen (CA) show significant clinical utility in diagnosing CRC and monitoring patients during the perioperative and postoperative phases [4, 5]. They may not offer superior specificity and early detection capabilities for AN. Up to now, CRC auxiliary diagnostic products based on molecular biology and technology have been marketed in China, including septin-9 methylation test based on blood samples and DNA methylation test based on fecal samples [6, 7]. The former does not have the advantage of early detection and has a significantly lower positive rate compared to fecal samples, whereas the latter's targets and technology are derived from the technology of marketed products in Europe and the United States and are based on basic research and clinical trials in European and American populations, with a lack of clinical data support in Asian populations, especially in China.

MiR-92a is a class of small molecule non-coding RNA, belonging to the miR-17-92 gene cluster on human chromosome 13, which promotes the proliferation and migration of CRC cells by inhibiting PTEN, KLF4, and downstream p21 gene [8-10], is a specific target for CRC, and has important value for early diagnosis, postoperative surveillance and prognosis of CRC [11]. Stool samples from patients with CRC or pre-cancerous high-risk lesions including polyps contained significantly higher levels of miR-92a than normal. Meanwhile, small RNAs can be effectively used as markers for CRC detection due to their stability and reproducibility in stool samples [12].

2.2 Fecal miR-92a detection technology

We has previously completed clinical trials in Shenzhen People's Hospital, Guangzhou Sun Yat-sen University Cancer Prevention and Treatment Centre and Tianjin People's Hospital for its miR-92a test based on stool samples. The product was approved for marketing by the National Medical Products Administration (NMPA) in March 2018 (National Machinery Registration Standard 20183400108) through the national innovative medical device special approval channel. A total of 1306 clinical subjects were enrolled in the pre-market clinical trial, including 340 cases of confirmed colorectal

cancer, 901 cases of confirmed non-colorectal cancer, including esophageal cancer, gastric cancer, hepatocellular cancer, pancreatic cancer, cholangiocarcinoma, oral cancer, gastritis, enteritis, appendicitis and peptic ulcer, 901 cases of patients with colorectal polyps and adenomas, as well as a control group of the normal population, and 65 cases of patients with post-surgery CRC.

The expression of miR-92a in the intestinal epithelial cells of CRC patients increases with the onset of the disease. Consequently, the detection of miR-92a in exfoliated intestinal epithelial cells obtained from fecal samples serves as an effective tool for identifying the risk of colorectal cancer (CRC), including pre-cancerous polyps, adenomas, and other related lesions. Stool miR-92a was detected by extracting total RNA from stool samples using a nucleic acid extraction reagent and performing miR-92a-specific RT-PCR reaction. The primers and probes in the reaction solution are designed to specifically bind to and amplify miR-92a and its reverse transcription product. Compared to colonoscopy, the fecal-based miR-92a assay demonstrates high sensitivity and specificity in detecting CRC and precancerous lesions like adenomas. It functions as a potent screening tool for identifying individuals at risk for CRC, and facilitates comprehensive screening and diagnosis of CRC and precancerous lesions, notably adenomas in their advanced stages.

2.3 Research content

The study was conducted at Shenzhen People's Hospital (SZH) (head unit), Shenzhen Bao'an District Hospital of Traditional Chinese Medicine (BAT) and Tianjin Nankai Hospital (NKH). 16,505 subjects were enrolled according to the inclusion and exclusion criteria. During the recruitment process, the researchers will fully introduce the content, risks and value of the study to the subjects. Subjects will sign an informed consent to participate in the study after they have been fully informed of the content, risks and benefits of the study and their questions have been answered. A short interview using the CRC risk questionnaire will be conducted and a stool sample collection kit will be given to the subjects. One stool sample will be collected according to the stool sample collection instructions and returned to the clinical site. The sample will be used for fecal-based miR-92a and FIT (9. Instructions for stool sample collection). Colonoscopy and/or pathology will be performed on subjects with positive fecal-based miR-92a and/or FIT. Some screen-negative individuals will also be randomly selected for colonoscopy and/or pathological examination. The study will analyze the performance of FIT, miR-92a and their combination in the screening of CRC and AA [advanced neoplasia (AN)] using colonoscopy and/or pathological findings as the gold standard.

3. Research target

To analyze the performance of FIT and fecal-based miR-92a and their combination for AN, and to evaluate the performance indicators and form an early screening and prevention strategy for people at average risk of CRC.

4. Research methods

4.1 Colorectal cancer risk questionnaire interview

After signing the informed consent, subjects will receive the CRC risk questionnaire. The content of the questionnaire will not be used as a basis or reference for the determination of the test results.

4.2 Fecal-based miR-92a test

4.2.1 Principle

The miRNA detection in fecal-based shedding of intestinal epithelial cells has good reproducibility and stability. MiR-92a is a molecular marker specific for CRC, and its level is elevated in CRC or precancerous lesions compared with that of normal subjects. MiR-92a can be used to evaluate the risk of CRC and precancerous lesions of the subjects through fecal samples.

4.2.2 Main components

Components	Number of tubes	Volume
MiR-92a RT-PCR solution	1	185 μl
MiR-92a PCR reaction solution	1	800 μl
MiR-92a RT-PCR primer	1	25 μl
MiR-92a PCR primers and probes	1	85 μl
Quality control product 1	1	20 μl
Quality control product 2	1	50 μl
Reverse transcriptase	1	33.5 μl
RNAase inhibitors	1	6.5 μl
Taq enzyme	1	15 μl
0.1% DEPC water	2	1200 μl

MiR-92a microRNA-92a, *RT-PCR* reverse transcription polymerase chain reaction, *PCR* polymerase chain reaction, *RNAase* ribonuclease, *DEPC* diethyl pyrocarbonate

4.2.3 Storage conditions and expiration dates

- The kits are valid for 12 months when stored at -20 ℃ and below;
- Try to avoid repeated freezing and thawing when using the kit, and the number of times of freezing and thawing shall not exceed 5;
- The date of manufacture and expiration are shown on product labels.

4.2.4 Procedure

- Fecal-based miR-92a test uses nucleic acid extraction reagents to extract total RNA from stool samples and perform miR-92a-specific RT-PCR on them.
- The primers and probes in the reaction solution can bind to and amplify miR-92a and its reverse transcription products.
- During the amplification process, once the probe complements and binds to the reverse transcription cDNA strand containing the miR-92a template, the fluorescent dye group positioned at the 5' end of the probe is cleaved by the Taq-enzyme, resulting in the release of fluorescent signals.
- The fluorescence signal released by the probe is monitored by a quantitative fluorescence PCR instrument in real time and a curve is generated to reflect the miR-92a content in the sample.
- Results determination:

  a. Positive: The Ct value of the sample to be tested is < 32.00.

  b. Negative: The Ct value of the sample to be tested is ≥ 32.00.

4.3 Fecal occult blood test

4.3.1 Principle

A fecal occult blood test looks for human hemoglobin (Hb) in a stool sample. Hb is a common target for fecal occult blood testing. Immunocolloidal gold-based fecal occult blood testing uses a monoclonal antibody to Hb as a double antibody sandwich to detect human Hb in stool samples. The FIT in this trial is an immunochromatographic assay for the detection of occult blood in fecal samples using two protein targets, hemoglobin and transferrin.

The Hb in the tested specimen first reacts with the gold standard anti-human hemoglobin monoclonal antibody A to form a complex, which moves upward chromatographically along the membrane by capillary action, and when it reaches

the detection line, it binds with the anti-human hemoglobin monoclonal antibody B encapsulated in the detection zone to form a red line, which is a positive result, and if the detection zone does not have a red line, then it is a negative result.

4.3.2 Main components of the kit

The test cards and strips consist of a sample pad, a gold standard pad, a nitrocellulose membrane, an absorbent paper, and a PVC plate. The gold standard pad has anti-human hemoglobin monoclonal antibody A, the nitrocellulose membrane which coated with anti-human hemoglobin monoclonal antibody B, and the quality control line which coated with sheep anti-mouse IgG antibody. Additional information on the FIT kit is detailed in the table below.

4.3.3 Storage conditions and expiration dates

- Storage conditions: Store at 4 ℃ to 30 ℃ in a dry place, protected from light and heat; do not freeze; take precautions to avoid heat, freezing and thawing in summer and winter.
- Expiration dates: 18 months. The reagent should be used within 1 hour after opening the inner package (humidity: 20 – 90%, temperature: 10 – 50 ℃).

4.3.4 Procedure

- Leave the reagent at room temperature for 30 min before use and return to room temperature (20 – 30 ℃). Do not open the inner package until it is ready for use, and use it within 1 hour after opening the inner package (humidity: 20 – 90%, temperature: 10 – 50 ℃).
- Add 1 ml of distilled won water to the fecal specimen and mix well for examination.
- Open the inner package, take out the reagent and put it on the countertop.
- Add 2 drops of diluted sample (approximately 80 ml) vertically to the reagent spiking tip and observe the result within 5 min, after which the result is invalid.
- Results determination:

  a. Positive: A red line appears at each of the test line (T line) and control line (C line).

  b. Negative: A red line appears at the C line only.

  c. Invalid: The absence of a red line at the C line indicates an operational error or reagent failure.

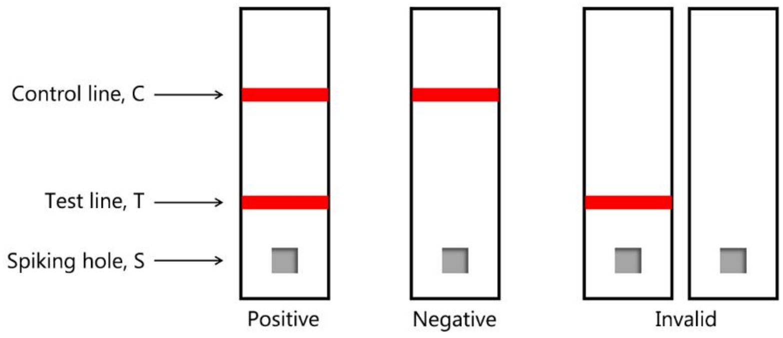

4.4 Colonoscopy

Principle: Colonoscopy uses a fiberoptic endoscope inserted through the anus to view the rectum, sigmoid colon, descending colon, transverse colon, ascending colon and ileocecum during entry and exit. Colonoscopy allows visualization of intestinal lesions and the microscopic removal or tissue sampling of polyps, such as progressive adenomas, and the removal of foreign bodies from the colorectum.

Procedure: Subjects are instructed to orally consume 3000 – 4000 ml of polyethylene glycol on the evening before the examination. During the examination, the endoscopist adheres to standardized protocols to ensure a comprehensive and clear visual of the anal canal, rectum, sigmoid colon, descending colon, transverse colon, ascending colon and ileocecal region. After microscopic lancing, clamping or scraping of the lesion, the tissue removed by the colonoscope is examined according to standard histopathological methods to determine the nature of the lesion.

4.5 Screening experiment design

In parallel experiments, if either of the tests (T) yielded positive results, the overall outcome (R) was considered positive, as demonstrated in equation (1). Parallel testing can enhance sensitivity and lower false negative occurrences, though at the expense of reduced specificity.

$$R_{+} = T_{1+} \cup T_{2+} \cup \ldots \cup T_{n+} \quad (1)$$

$$SN(Tc) = \sum_{i=1}^{S} SN(Ti) + \prod_{i=1}^{S} SN(Ti) - \left(\sum_{i=1}^{S} SN(Ti)\right)^2 + \sum_{i=1}^{S} (SN(Ti))^2 \quad (2)$$

$$SP(Tc) = \prod_{i=1}^{S} SP(Ti) \quad (3)$$

When the number of tests S=2, the formula can be substituted as:

$$SN(Tc) = SN(T_1) + SN(T_2) - SN(T_1) \times SN(T_2)$$

$$= SN(T_1) + SN(T_2) \times [1 - SN(T_1)] \quad (4)$$

$$SP(Tc) = SP(T_1) \times SP(T_2) \quad (5)$$

5. Study population

5.1 Enrolment of subjects

The target population for this study was the average risk group for CRC aged 30 – 75 years. Subjects will be recruited based on the inclusion and exclusion criteria. Subjects will be excluded if they are recruited but meet the exclusion criteria. The target population is divided into age groups of 5 years. Laboratory testing will be initiated as soon as the subject has been successfully recruited and has provided a satisfactory stool sample. The researchers will monitor the progress of the subjects and the results of the laboratory tests in real time and will count the number of enrolments and fecal-based miR-92a positivity in time to determine whether to terminate the enrolment early.

5.2 Inclusion criteria

- Age between 30 and 75;
- Able to provide a stool sample and agree to undergo colonoscopy;
- Agree to participate in the study and sign an informed consent.

5.3 Exclusion criteria

- History of colorectal cancer or treatment with colorectal cancer drugs or surgery;
- History of advanced adenomas or colorectal polyps;
- Patients with severe mental retardation or speech communication disorder, severe heart, brain, lung disease or liver or kidney dysfunction;
- Patients with severe ulcerative colitis;
- Patients with hereditary CRC syndromes (e.g., Lynch syndrome, FAP);
- Subjects who request to withdraw from this study;
- Subjects who are deemed unsuitable for this study by the investigator after thorough discussion.

5.4 Removal criteria

- Samples that meet any of the following criteria are considered ineligible and should be excluded from the study; subjects whose samples have been excluded may continue to participate in the study if they have been resampled to obtain an eligible sample:

- ✓ Insufficient sample size or visually significantly less than the sample size required for the program;
- ✓ The sample is contaminated;
- ✓ Watery stools;
- ✓ A sample that is loose stool and does not contain any formed portion;
- ✓ Mucus or pus stools;
- ✓ Bloody stools;

- Subjects with failed sample unwilling to cooperate with resampling;
- Subjects with inadequate bowel preparation or where the colonoscope does not reach the blind part of the model;
- Samples for which laboratory testing cannot be completed for unknown reasons;
- Subjects with an unclear diagnosis or incomplete case history;
- Subjects who are considered unsuitable for the study after full discussion with the investigator.

5.5 Sample size considerations

The required sample size for the test is based on the following assumptions:

The ratio of AA to CRC in general risk population is 10:1;

Among the population aged 30 – 75, approximately 30 out of every 10,000 people are patients with CRC and 300 out of every 10,000 people are patients with AA;

The sensitivity and specificity of fecal-based miR-92a detection for CRC are 90% and 90%, respectively. The sensitivity and specificity of fecal-based miR-92a detection for AA are 80% and 90%, respectively;

The sample dropout rate is 5%.

According to the Buderer formula [13], the required sample size is estimated as follows:

Step1: Specifications

SN: sensitivity; SP: specificity; 1-α: confidence level; $Z_{1-\alpha/2}$: standard normal deviation (when α = 0.05, $Z_{1-\alpha/2}$ = 1.96); W: allowable error, set to 0.09 here; Prevalence: prevalence rate, which is the ratio of positive cases in all samples. We set the prevalence rate for CRC to be 0.3% and the prevalence rate for AA to be 3%.

Step2: Calculate the number with disease, TP + FN

$$TP + FN = {Z_{1-\alpha/2}}^2 \frac{SN(1 - SN)}{W^2}$$

Step3: Calculate the sample size required for sensitivity, N_1

$$N_1 = \frac{TP + FN}{P}$$

For CRC, $N_1 = 14{,}228$; for AA, $N_1' = 2{,}529$

Step4: Calculate the number without disease

$$FP + TN = Z_{1-\alpha/2}{}^{2}\frac{SP(1 - SP)}{W^{2}}$$

Step5: Calculate the sample size required for specificity, N_2

$$N_2 = \frac{FP + TN}{1 - P}$$

For CRC, $N_2 = 43$; for AA, $N_2' = 78$

Step6: Select the final sample size, N

$$N = 14{,}228$$

In summary, considering a 5% dropout rate, the required sample size is 14,975. To ensure sufficient sample size after excluding unqualified samples, 15,586 individuals were ultimately included in this study.

6. Research program design and processes

6.1 Subjects enrolled

Subject enrolment will be initiated after the project has been approved by the ethical review committee. The researchers will determine whether to recruit subjects based on the inclusion and exclusion criteria, explain the study in detail to those who meet the enrolment criteria and are interested in participating in the trial, and answer all questions raised by the subjects to ensure that they are fully aware of the content of the study, the risks, benefits and other relevant information. The subject signs the informed consent to participate in the study. Subjects have the right to withdraw from the study at any stage after successful recruitment. Subjects may contact the study leader to inquire about test results or to request withdrawal.

The researcher will use the CRC risk questionnaire to interview the subjects after they have signed the informed consent to participate in the study. One copy of the informed consent will be retained by the subject after signing, and the other copy will be kept on file together with the subject's information and code. The subject code will be used as the unique identification number of the subject, which will be used to identify the samples, laboratory test data, and summarize the required information to meet the needs of information query and verification. Stool samples will be taken from the subjects.

6.2 Sample reception and storage

Generally, the FIT and miR-92a will be conducted on the same day. Researchers should mix the qualified fecal samples in the sample tubes within 12 h after receiving the samples, and then divide them into five 2 ml centrifuge tubes, each tube 0.3 – 0.5 g, and freeze them in a -80 ℃ refrigerator. If the sample cannot be packaged in a timely manner, the sample tube should be temporarily stored in a 4 ℃ refrigerator and the sample packaging and freezing should be completed within 24 h.

6.3 Fecal-based miR-92a test

One centrifuge tube of the subject's fecal samples will be used for fecal-based miR-92a testing, and the rest of the subject's fecal samples will continue to be frozen at -80 ℃ as a backup.

6.4 Fecal occult blood test

Allow the reagent chamber to stand at room temperature for 30 min before use. Open the foil pouch of FIT test, remove the colloidal gold test card and mark the sample number. Using a proctor, pick up the stool sample from multiple points (not less than 6 points) on the surface of the stool sample into the sample tube containing the diluent, mix well by inverting the sample, add 2 – 3 drops of liquid to the colloidal gold test card and observe the "C" and "T" lines within 5 min. FIT testing is completed immediately upon receipt of the sample.

6.5 Colonoscopy and/or pathology

Subjects are instructed to orally consume 3000 – 4000 ml of polyethylene glycol on the evening before the examination. During the examination, the endoscopist adheres to standardized protocols to ensure a comprehensive and clear visual of the anal canal, rectum, sigmoid colon, descending colon, transverse colon, ascending colon and ileocecal region. After microscopic lancing, clamping or scraping of the lesion, the tissue removed by the colonoscope is examined according to standard histopathological methods to determine the nature of the lesion.

6.6 Follow-up management

According to the protocol design and workflow, the study will be conducted in subjects who are positive for fecal-based miR-92a and/or FIT, and a random sample of subjects who are negative for tests will be randomly selected for colonoscopy, and subjects will be followed up until completion of colonoscopy. Individuals who have undergone

screening and require confirmatory testing should promptly schedule a colonoscopy and engage in health education sessions without delay.

6.6.1 Subjects tested positive for fecal samples

The researchers actively followed up with positive subjects, i.e., fecal miR-92a positive subjects, FIT positive subjects, miR-92a + FIT positive subjects, and provided education on bowel health and CRC prevention while facilitating the timely completion of colonoscopy.

The researchers counted the results of fecal samples in batches and contacted the subjects with positive fecal miR-92a and/or FIT tests to follow up with them to receive the colonoscopy and to make an appointment for the colonoscopy. After confirming the subject's acceptance of the colonoscopy and setting a date for the colonoscopy, the researcher contacted the subject again to follow up with him/her to learn about the colonoscopy precautions and to remind him/her to pick up the medications and materials needed for the bowel preparation. The researcher retrieved the colonoscopy report and pathology results in a timely manner after the subject completed the colonoscopy.

6.6.2 Subjects tested negative for fecal samples

The researchers actively followed up with randomly selected subjects under the following three conditions and provided education on bowel health and bowel cancer prevention while promoting timely completion of colonoscopies:

- A comparable number of fecal miR-92a-positive subjects were randomly chosen from among the fecal miR-92a-negative subjects.
- A comparable number of FIT-positive subjects were randomly chosen from among the FIT-negative subjects.
- A comparable number of fecal miR-92a & FIT-positive subjects were randomly chosen from among the fecal miR-92a & FIT-negative subjects.

Random method: Develop a Microsoft Excel worksheet to systematically categorize and enumerate all fecal test-negative subjects who have successfully undergone fecal sample testing but have not been randomized. Specifically, segregate the subjects into three distinct groups: those who tested negative for fecal miR-92a, those who tested negative for FIT, and those who tested negative for both fecal miR-92a and FIT. Number each subject within their respective group, and categorize them further based on age groups, with a distinct consideration for gender. Populate column A with the sequential subject numbers for each age-gender group. Utilize the Random function in column B, executing the command by pressing the Enter key, and subsequently generate the data columns by dragging down the cells. Select the

corresponding subject numbers for follow-up in ascending order to ensure a systematic and unbiased approach for further analysis.

Random example: A cohort of 200 subjects, who had undergone stool sample testing but had not been randomized, comprised the study population. This group included 120 males and 80 females. Notably, within the 51 – 55 age bracket of male subjects, 40 individuals were evaluated, among whom 3 tested positives for miR-92a. Subsequently, the researchers adopted a randomized approach to select 3 of the remaining 37 male subjects within this age group, who had negative miR-92a test results, for active follow-up and colonoscopy. The methodology employed for this selection and subsequent colonoscopy procedure is outlined below.

- Create a new Microsoft Excel worksheet and enter the subject numbers of fecal miR-92a test-negative male subjects in the 51-55 age group in column A, e.g., SZH00051-SZH00087.
- In cell B1 enter the function: = RAND ().
- After clicking on the model to execute the function, drop down the cell and generate random data.
- Select the data cells in column B, right-click the mouse and select "Sort" in the "ascending".
- By expanding the selected region, the data in column A can be redistributed randomly.
- Select the first three rows of the worksheet, select the toolbar in the "data" menu in the "combination" option, read the number obtained that is randomly selected subjects.
- Initiate a follow-up telephone call with three randomly selected subjects to follow up on the completion of the colonoscopy. If any of the subjects refused to undergo colonoscopy, the randomized follow-up subjects were deferred.

6.7 Information entry and collation

Information including CRC risk questionnaire, subject code, age, gender, fecal-based miR-92a test results, FIT test results, colonoscopy and pathology results, and hospital discharge diagnosis or medical records will be aggregated by the investigator into the research database after removing personally identifiable information. Subject enrolment, sample testing and compilation of information will be conducted simultaneously to allow the investigator to provide timely feedback on subsequent enrolment based on sample collection and testing. Personal identification information includes, but is not limited to, the subject's name, ID card number, social security account number, driver's license number, clinic number, hospitalization number and any other information that can directly or indirectly identify the subject.

6.8 Research materials and equipment

The company provides stool sample collection kits, nucleic acid extraction reagents, fecal-based miR-92a test reagents, FIT test reagents, and any other materials necessary for the collection of stool samples from subjects and for laboratory testing. Clinical facilities conducting the trial will provide space, equipment and materials for colonoscopy and histopathology examinations.

6.9 Back-up sample storage

Backup samples will be kept in a refrigerator at −80 ℃ in the clinical medical research center for future traceability and verification, and the samples will be kept for no more than 2 years.

7. Data analysis

7.1 Test termination points

7.1.1 Relevant definitions

The main objective of the study was to examine the performance of FIT, miR-92a, and FIT + miR-92a for AN. Colonoscopy and histopathology were used as the gold standard to determine the classification of the subject.

**Screening test**	**Colonoscopy and histopathology**	
	**Patients**	**Nonpatients**
Positive	True positive (TP)	False positive (FP)
Negative	False negative (FN)	True negative (TN)

When counting the performance of different screening protocols for CRC, subjects with a positive screening test result and a confirmed colonoscopy diagnosis of CRC were counted as true positives, otherwise they were counted as false positives, and subjects with a negative screening test and a confirmed colonoscopy diagnosis of a non-colorectal cancerous lesion, such as a normal, inflamed, polyps, advanced adenoma, or other non-colorectal cancerous lesion were counted as true negatives, otherwise they were counted as false negatives.

When counting the performance of different screening protocols for AN (CRC and AA), a positive screening test with a confirmed colonoscopy diagnosis of CRC or AA was counted as a true positive, otherwise it was counted as a

false positive, and a negative screening test with a confirmed colonoscopy diagnosis of a normal, inflamed, polyps, or other non-colorectal cancerous lesion was counted as a true negative, otherwise it was counted as a false negative. Specific screening and analysis performance indicators are calculated as follows:

- Sensitivity (SN): also known as true positive rate (TPR), defined as true positive/ (true positive + false negative) × 100%;
- Specificity (SP): also known as true negative rate (TNR), defined as true negative/ (true negative + false positive) × 100%;
- Accuracy (ACC): defined as (true positive + true negative)/ (positive + negative) × 100%;
- Positive predictive value (PPV): defined as true positive/ (true positive + false positive) × 100%;
- Negative predictive value (NPV): defined as true negative/ (true negative + false negative) ×100%.

7.1.2 Categorization of participants based on colonoscopy and histopathology

Colonoscopy and/or histopathological findings were classified as AA if they met any of the following criteria:

- Colonoscopy and/or pathology for carcinoma in situ;
- Serrated polyps with maximum size ≥ 1 cm on colonoscopy and/or pathology;
- Tubular/chromosomal adenoma (≥ 25% choriocapillaris) of any size on colonoscopy and/or pathology;
- Tubular adenoma with high-grade intraepithelial neoplasia of any size on colonoscopy and/or pathology;
- Tubular adenoma, low-grade intraepithelial neoplasia, maximum size ≥ 1 cm on colonoscopy and/or pathology;
- A colonoscopy and/or pathology that did not meet any of the above criteria and did not meet the criteria for cancer was classified as normal or polyp or other, as determined by the colonoscopy and/or pathology.

Colonoscopy and histopathological findings of cancer will be staged according to TNM staging criteria as follows:

		T	N	M
0	0	Tis	N0	M0
I	I	T1/T2	N0	M0
II	II	T3/T4	N0	M0
	IIA	T3	N0	M0
	IIB	T4a	N0	M0
	IIC	T4b	N0	M0
III	III	Tis-T4	N+	M0
	IIIA	T1 – T2	N1	M0
		T1	N2a	M0
	IIIB	T3 – T4a	N1	M0
		T2	N2a	M0
		T1-T2	N2b	M0
	IIIC	T4a	N2a	M0
		T3 – T4a	N2b	M0
		T4b	N1 – N2	M0
IV	IV	Tis-T4	N0 – N2	M+
	IVA	Tis-T4	N0 – N2	M1a
	IVB	Tis-T4	N0 – N2	M1b

7.1.3 Endpoint

The objective of the study is to analyze the performance of FIT, miR-92a and their combination for the detection of AN, evaluating performance indicators such as sensitivity, specificity, predictive values, diagnostic accuracy, likelihood ratios, and overall compliance rate, to formulate a strategy for early screening and prevention of CRC for an average-risk population. Once the project is initiated, the researchers will monitor the progress of enrolment, results of miR-92a and FIT, and colonoscopy and pathology results in real time, and will determine whether to terminate enrolment early based on the number of enrolments and fecal-based miR-92a positivity rate.

Data summaries for all subjects will be summarized in a frequency table:

Test result	Colonoscopy and/or histopathology			Total
	CRC	AA	NEG	
Positive	a	b	c	a + b + c
Negative	d	e	f	d + e + f
Total	a + d	b + e	c + f	N

CRC colorectal cancer, *AA* advanced adenomas, *NEG* negative (including polyps, other non-colorectal cancerous lesion and no findings)

(1) The sensitivity and specificity will be calculated as:

- xx test sensitivity for subjects with CRC = a/ (a + d)
- xx test sensitivity for subjects with AA = b/ (b + e)
- xx test sensitivity for subjects with AN = (a + b)/ (d + e)
- xx test specificity for subjects with negative findings = f/ (c + f)

(2) The PPV and NPV will be calculated as:

- PPV (CRC) = Probability of having CRC given a positive xx test result = a/ (a + b + c)
- PPV (AA) = Probability of having AA given a positive xx test result = b/ (a + b + c)
- PPV (AN) = Probability of having AN given a positive xx test result = (a + b)/ (a + b + c)
- NPV = Probability of having negative findings on colonoscopy given a negative xx test result = f/ (d + e + f)

(3) The LRs will be calculated as:

- PLR (CRC) = P (Test positive | CRC)/P (test positive | No CRC) = a/ (b + c)
- PLR (AA) = P (Test positive |AA)/P (test positive | No AA) = b/ (a + c)
- PLR (AN) = P (Test positive | AA or CRC)/P (test positive | No AA nor CRC) = (a + b)/c
- NLR (NEG) = P (Test negative | NEG)/P (test negative|AA or CRC) = f/ (d + e)

7.2 Analysis of clinical outcomes

The main indicators for the analysis of clinical results were the sensitivity, specificity, predictive values, diagnostic accuracy and likelihood ratios of FIT, miR-92a, and their combination in screening AN, using the results of colonoscopy and histopathology examination as the gold standard. The data were analyzed using SAS, SPSS, Stata or other commonly used clinical research data analysis software to obtain the best cut-off value.

7.3 Other research statistics

The other research outcomes encompass the detection rate of AN by FIT, miR-92a, and their combination.

7.4 Statistical methods

A variety of categorical data analysis methods will be used to analyze the results from the miR-92a, FIT and FIT + miR-92a results. Binomial proportions and exact 95% CI summarize sensitivity and specificity across categories. According to the study design, FIT and miR-92a from the same subjects were considered as a paired design, and the exact McNemar's test was used to compare the differences of DR, PR, Se and Sp between these two groups. Given the susceptibility of PPV to confounding factors like prevalence and subject characteristics, the GEE (Generalized Estimating Equations), which is based on McNemar's test, was utilized to compare the differences in PPV between the two groups.

8. Ethical issues

8.1 Risk analysis

8.1.1 Subject risks

Stool samples will be collected non-invasively, using a stool sample kit that allows subjects to collect stool samples in a self-contained and convenient manner. The process of stool sample collection may be psychologically uncomfortable for the subject, but there is no risk to the subject's health. The fecal-based miR-92a test and FIT are performed in the laboratory by professional technicians. There is no risk to the subject's health from the sample testing process.

Subjects will be asked to undergo colonoscopy when they have a positive result for fecal-based miR-92a or FIT or when they have a negative result for fecal-based miR-92a or FIT but are contacted by the investigator on a random basis. Subjects will be offered the choice of a plain colonoscopy or a painless colonoscopy. Subjects will be asked to complete bowel preparation prior to the colonoscopy and may experience discomfort because of evacuating the bowel after taking oral bowel cleansing medications. General colonoscopy is invasive and may pose a risk of bowel perforation or bleeding. Subjects may briefly experience bloating after the colonoscopy due to inflation of the bowel during the procedure.

Painless colonoscopy is invasive and may pose a risk of perforation or bleeding. Subjects may briefly experience bloating after the colonoscopy due to inflation of the bowel during the procedure. A painless colonoscopy will be

conducted under general anesthesia before the procedure begins and will continue while the subject remains under general anesthesia. This may carry a risk of allergic reactions to anesthetics and other anesthesia-related risks for the subjects. Additionally, there may be restrictions on activities such as driving, cycling or working at heights on the day following the painless colonoscopy procedure.

8.1.2 Information confidentiality

The personal information and privacy of subjects involved in this trial will be protected and will not be disclosed. Any personally identifiable information of the subjects will be stored on a password-protected carrier that is accessible only to investigators at the participating clinical site where the subject is enrolled. Personally identifiable information includes, but is not limited to, the subject's name, identification number, social security account number, driver's license number, clinic number, hospitalization number, and any other information that directly or indirectly identifies the subject as an individual. Subject sample numbers will be assigned by the enrolled participating clinical site.

8.1.3 Subject benefits

Through enrolment in the study, subjects will receive 100 RMB as compensation for travelling expenses. Subjects will be offered a free fecal-based miR-92a test and FIT with result interpretation by a professional technician and possible follow-up management to improve knowledge of CRC prevention and early detection of possible risk of intestinal lesions.

Subjects will be offered a free colonoscopy if they have a positive fecal-based miR-92a test or FIT. If lesions are found, samples of the lesions will be taken for histopathology examination. Subjects can choose to undergo either plain or painless colonoscopy. The cost of colonoscopy (plain or painless) and histopathology examination will be borne by the company.

Subjects will be offered free colonoscopy when they have a negative fecal-based miR-92a test or a negative FIT test but are contacted by the investigator at random. If microscopic lesions are identified, they will be sampled for histopathology examination. Subjects may choose to undergo either a plain colonoscopy or a painless colonoscopy. The cost of colonoscopy (plain or painless) and histopathology examination will be borne by the company.

Subjects will not be offered a free colonoscopy if they have a negative fecal-based miR-92a test or a negative FIT test but are not contacted by the investigator at random.

8.1.4 Explanation of test safety

The use of fecal samples in this study does not pose any threat to the safety of the subjects.

8.2 Informed consent

The informed consent was strictly formulated in accordance with the relevant regulations and was submitted to the ethics review board for approval. Subjects have the right to review and comprehend the terms and conditions, as well as to ask questions regarding any of the terms and conditions. A copy of the informed consent will be given to the participant after he/she fully understands the content, risks and value of the study and signs it before enrolment in the study.

8.3 Data reliability

In this study, fecal-based miR-92a test and FIT will be done by professional technicians to ensure the accuracy of the sample laboratory test results. Colonoscopy will be performed by experienced endoscopists in strict accordance with the standard protocols for colonoscopy to avoid bias in the test results caused by human intervention. Subjects' colonoscopy and histopathological results will be entered and analyzed using clinical research database tools, so that the results will not be biased due to the researcher's subjective awareness.

8.4 Project management

Subject recruitment, sample collection and laboratory testing, colonoscopy and histopathology examination, and information collation will be controlled by the project investigators to ensure the proper functioning of the project. Confidentiality of information will be followed up and monitored by the head of the clinical site to ensure the proper conduct of the trial.

8.5 Ethics review committee

- Ethics review committee of Shenzhen People's Hospital (head unit) (Reference ID: SYL-202123-03);
- Ethics review committee of Shenzhen Bao'an District Hospital of Traditional Chinese Medicine (Reference ID: SJ-2021-001-04);
- Ethics review committee of Tianjin Nankai Hospital (Reference ID: NKYY_YX_IRB_2021_002).

9. Instructions for stool sample collection

(1) Stool sample collection kit contains: sampling instructions, ice pack, sample tube, stool collection paper, self-sealing bag, insulated bag and disposable gloves.

(2) The day before sampling, open the sample collection kit and take out the ice pack; open the water spout of the ice pack and fill it with tap water, gently squeeze out the excess air and close the water spout, then shake it well and put it into the freezer for more than 5 h. Women should avoid collecting fecal samples during their menstruation period, but can collect fecal samples before or after it.

(3) When picking stool, open the package of plastic stool paper, tear off the protection paper of the adhesive strip on both sides of the stool paper, use the double-sided adhesive strip to fix the stool paper on both sides of the toilet ring, and make the middle of the stool paper downward to form a curvature. The plastic stool paper was used to collect the samples and avoid contamination with toilet water.

(4) Defecation was performed at the curve of the stool collection paper. After completing defecation, the subject should wear disposable gloves, take out the sample tube and fill in the subject's name, subject number, and sampling time (e.g., 9:00 AM, May 18, 2021) in regular script on the label of the tube. Use the feces sampling spoon to scrape the feces from multiple points on the surface of the feces for no less than 6 times, with each scraping being the size of a broad bean and placing them at the bottom of the sample tube. If there are visibly identifiable food residues in the feces, such as watermelon seeds or corn kernels, the subject should avoid these areas when sampling and prevent food residues from entering the sample tube.

(5) Each time, a feces sample of no less than 10 g should be collected. Multiple samplings can be done to ensure that the total sample quantity meets the requirement, which is visually confirmed by ensuring that the sample volume exceeds the 10mL mark when placed at the bottom of the sample tube. When collecting the feces, the subject should ensure that the feces sample is formed or semi-formed, sufficient in quantity, and free from contamination.

(6) After completing the sample collection, the sample tube containing the fecal sample was screwed with a tight cap and placed in a self-sealing bag.

(7) After completing the above steps of fecal sample collection, the double-sided adhesive strips on both sides of the fecal paper were removed from the toilet rim and discarded into the toilet bowl for flushing.

(8) Upon completion of the fecal sample collection, the frozen ice pack is placed in an insulated bag along with the self-sealing bag with the built-in fecal sample tube and returned to the clinical agency field staff on the same day.

The process is illustrated below:

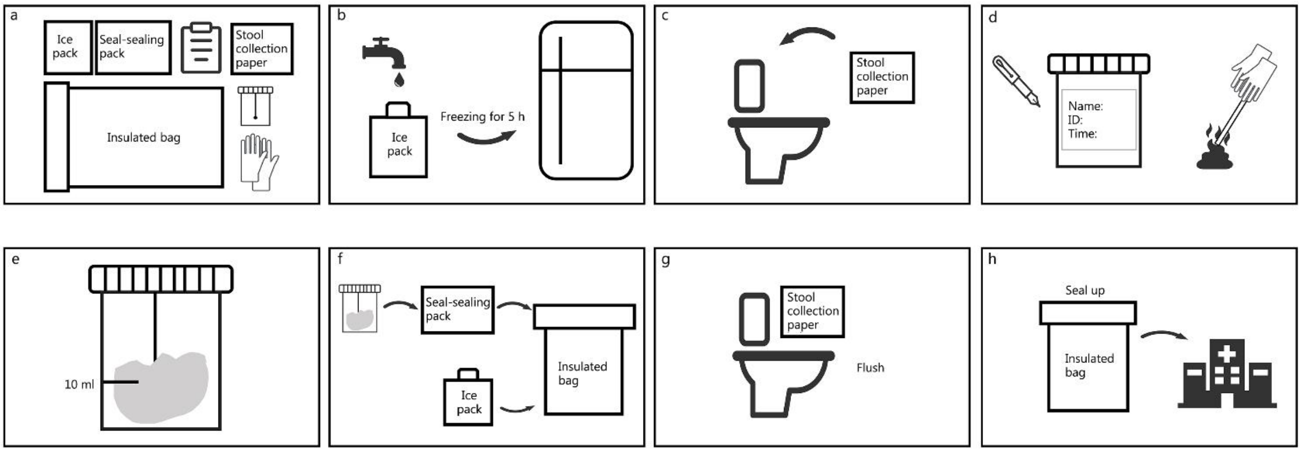

10. References

1. Sung H, Ferlay J, Siegel RL, Laversanne M, Soerjomataram I, Jemal A, et al. Global Cancer Statistics 2020: GLOBOCAN estimates of incidence and mortality worldwide for 36 cancers in 185 countries. CA Cancer J Clin. 2021;71(3):209-49.
2. Chang WJ, Ren L, Xu JM. 2019 Updated interpretation of the guidelines for the diagnosis and treatment of colorectal cancer liver metastases. Chin J Basic Clinic Surg. 2019;26:907-11.
3. Wolf AMD, Fontham ETH, Church TR, Flowers CR, Guerra CE, LaMonte SJ, et al. Colorectal cancer screening for average-risk adults: 2018 guideline update from the American Cancer Society. CA Cancer J Clin. 2018;68(4):250-81.
4. Dalamaga M, Karmaniolas K, Kontos F, Migdalis I, Dionyssiou-Asteriou A. Clinical study on the serum carcinoembryonic antigen, CA 19-9, CA 50 and α-fetoprotein levels in patients with myelodysplastic syndromes. Leuk Lymphoma. 2006;47(9):1782-7.
5. Oh T, Kim N, Moon Y, Kim MS, Hoehn BD, Park CH, et al. Genome-wide identification and validation of a novel methylation biomarker, SDC2, for blood-based detection of colorectal cancer. J Mol Diagn. 2013;15(4):498-507.
6. Tóth K, Sipos F, Kalmár A, Patai AV, Wichmann B, Stoehr R, et al. Detection of methylated SEPT9 in plasma is a reliable screening method for both left- and right-sided colon cancers. PLoS One. 2012;7(9):e46000.
7. Imperiale TF, Ransohoff DF, Itzkowitz SH, Levin TR, Lavin P, Lidgard GP, et al. Multitarget stool DNA testing for colorectal-cancer screening. N Engl J Med. 2014;370(14):1287-97.
8. Ke TW, Wei PL, Yeh KT, Chen WT, Cheng YW. MiR-92a promotes cell metastasis of colorectal cancer through PTEN-mediated PI3K/Akt pathway. Ann Surg Oncol. 2015;22(8):2649-55.
9. Zhang GJ, Li LF, Yang GD, Xia SS, Wang R, Leng ZW, et al. MiR-92a promotes stem cell-like properties by

activating Wnt/β-catenin signaling in colorectal cancer. Oncotarget. 2017;8(60):101760-70.

10. Lv H, Zhang Z, Wang Y, Li C, Gong W, Wang X. MicroRNA-92a promotes colorectal cancer cell growth and migration by inhibiting KLF4. Oncol Res. 2016;23(6):283-90.

11. Thomas AM, Manghi P, Asnicar F, Pasolli E, Armanini F, Zolfo M, et al. Metagenomic analysis of colorectal cancer datasets identifies cross-cohort microbial diagnostic signatures and a link with choline degradation. Nat Med. 2019;25(4):667-78.

12. Wu CW, Ng SS, Dong YJ, Ng SC, Leung WW, Lee CW, et al. Detection of miR-92a and miR-21 in stool samples as potential screening biomarkers for colorectal cancer and polyps. Gut. 2012;61(5):739-45.

13. Buderer NM. Statistical methodology: I. Incorporating the prevalence of disease into the sample size calculation for sensitivity and specificity. Acad Emerg Med. 1996;3(9):895-900.
