# Supplementary material for: Diagnostic efficacy of fecal-based miR-92a for advanced colorectal neoplasia: a prospective multicenter screening trial
Source: Mil Med Res. 2025 Jun 13;12:30. doi: 10.1186/s40779-025-00613-3 (PMC12164163; doi:10.1186/s40779-025-00613-3)
Supplement: Supplementary file 2 — Additional file 2. Table S1 FIT Brands, Hemoglobin Detection Level and Stability and Storage Information. Table S2 The definitions of outcomes in this study. Table S3 Survey questions and responses provided by participants at the time of enrollment. Table S4 Demographics and socioeconomic status for participants who were enrolled but did not complete all study requirements (withdrawn cohort) compared to those who completed all study requirements (eligible cohort). Table S5 Symptoms or signs of gastrointestinal disorders or psychological issues for participants who were enrolled but did not complete all study requirements (withdrawn cohort) compared to those who completed all study requirements (eligible cohort) [n (%)]. Table S6 Demographic and socioeconomic risk factors and symptoms or signs of gastrointestinal disorders or psychological issues for screening-positive (FIT positive and/or miR-92a positive) participants who were enrolled but did not complete all study requirements (withdrawn cohort) compared to those who completed all study requirements (eligible cohort). Table S7 Demographic and socioeconomic risk factors and symptoms or signs of gastrointestinal disorders or psychological issues for screening double-negative (FIT negative and miR-92a negative) participants who were enrolled but did not complete all study requirements (withdrawn cohort) compared to those who completed all study requirements (eligible cohort). Table S8 Logistic regression model to assess the impact of dropout on the FIT, miR-92a and FIT + miR-92a sensitivity and specificity [% (95% CI)]. Table S9 Positive rate by miR-92a test between different groups. Table S10 Compliance rates among high-risk population between different groups. Table S11 FIT, miR-92a and FIT + miR92a screening for AN. Table S12 Sensitivity and specificity of the miR-92a and FIT + miR-92a test by age [% (95% CI)]. Table S13 Sensitivity and specificity by demographic and socioeconomic risk factors: sex, smoking, hist [file 40779_2025_613_MOESM2_ESM.pdf]

Table S1 FIT brands, hemoglobin detection level and stability and storage information

**Product name**	**Manufacturer**	**Collection device**	**Hemoglobin detection level**	**Stability, storage of sample**
Diagnostic Kit for Fecal Occult Blood (Colloidal Gold)	Qingdao Hantang	Liquid-vial	200 ng/ml	12 h at room temperature or 3 d at 2 – 8 ℃

FIT immunochemical fecal occult blood test

Table S2 The definitions of outcomes in this study

**Outcomes**	**Definition**
Positive rate	Proportion of individuals who test positive out of the total number of individuals screened
Compliance rate	Proportion of individuals who comply with or undergo colonoscopy after being identified as needing further diagnostic evaluation, which based on screening test results
Detection rate	Number of individuals who test positive and are later confirmed to have AN (CRC + AA), divided by the total number of individuals who completed colonoscopies
Sensitivity	Proportion of individuals with AN (CRC + AA) who test positive on the screening test. Sensitivity = (True positives)/ (True positives + False negatives) × 100%
Specificity	Proportion of individuals without AN (CRC + AA) who test negative on the screening test. Specificity = (True negatives)/ (True negatives + False positives) × 100%
Positive predictive value	Proportion of those with a positive screening test who have AN (CRC + AA) confirmed by colonoscopy. PPV = (True positives)/ (True positives + False positives) × 100%
Negative predictive value	Proportion of those with a negative screening test who do not have AN (CRC + AA) confirmed by colonoscopy. NPV = (True negatives)/ (True negatives + False negatives) × 100%
Accuracy	Proportion of participants correctly predicted by the screening test
Positive likelihood ratios	The ratio of the probability of a positive test result in the presence of AN (CRC + AA) to the probability of a positive test result in the absence of AN (CRC + AA). PLR = True positive rate/False positive rate = Sensitivity/ (1 – Specificity)
Negative likelihood ratios	The ratio of the probability of a negative test result in the presence of AN (CRC + AA) to the probability of a negative test result in the absence of AN (CRC + AA). NLR = False negative rate/True negative rate = (1 – Sensitivity)/Specificity
Youden index	Evaluation metrics of performance that combine sensitivity and specificity. Youden index = Sensitivity + Specificity – 1

AN advanced neoplasia, *CRC* colorectal cancer, *AA* advanced adenomas, *PPV* positive predictive value, *NPV* negative predictive value, *PLR* positive likelihood ratios, *NLR* negative likelihood ratios

Table S3 Survey questions and responses provided by participants at the time of enrollment

**Survey questions**	***n* (%)**
Have you ever smoked? (*n* = 15,586)	
Yes	1644 (10.55)
No	13,907 (89.23)
Unknown	35 (0.22)
Have you ever had a FOBT test within 5 years? (*n* = 15,586)	
Yes (FIT)	5 (0.03)
Yes (gFOBT)	115 (0.74)
No	15,148 (97.19)
Unknown	318 (2.04)
What were the results of the FOBT test? (*n* = 120)	
Positive	97 (80.83)
Negative	22 (18.33)
Unknown	1 (0.84)
Have you ever had a colonoscopy within 5 years? (*n* = 15,586)	
Yes	488 (3.13)
No	15,098 (96.87)
What were the results of the colonoscopy? (*n* = 488)	
Others	98 (20.08)
Normal	389 (79.71)
Uncompleted	1 (0.21)
Have you ever had cancer[*]? (*n* = 15,586）	
Yes	61 (0.39)
No	15,525 (99.61)
Have you ever had a first-degree relative※ who has had colorectal cancer? (*n* = 15,586)	
Yes	155 (0.99)
No	15,431 (99.01)
Who were the first-degree relatives※ who have had colorectal cancer? (*n* = 155)	
Parents	120 (77.42)
Siblings	33 (21.29)
Parents and siblings	2 (1.29)

[#]Including current smokers and ex-smokers. [*]Individual history of various cancers including colorectal cancer. ※First-degree relative (parent, sibling, child).

CRC colorectal cancer, *AA* advanced adenomas, *FIT* immunochemical fecal occult blood test, *FOBT* fecal occult blood test

Table S4 Demographics and socioeconomic status for participants who were enrolled but did not complete all study requirements (withdrawn cohort) compared to those who completed all study requirements (eligible cohort)

**Characteristics**	**Eligible cohort (*n* =3480)**	**Withdrawn cohort (*n* = 12,106)**	***P*-value**
Age (year, mean ± SD)	53.9 ± 10.8	51.1 ± 11.9	< 0.001
Sex [*n* (%)]			< 0.001
Male	1461 (41.98)	5611 (46.35)	
Female	2019 (58.02)	6495 (53.65)	
Smoking [*n* (%)]			0.018
Previous smoker	386 (11.09)	1251 (10.34)	
Non-smoker	3080 (88.51)	10,834 (89.49)	
Unknown	14 (0.40)	21 (0.17)	
Diabetes history [*n* (%)]			0.062
Yes	46 (1.32)	114 (0.94)	
No	3434 (98.68)	11,992 (99.06)	
Cancer history other than CRC [*n* (%)]			0.367
Yes	9 (0.26)	46 (0.38)	
No	3471 (99.74)	12,060 (99.62)	
Family history of CRC※ [*n* (%)]			< 0.001
Yes	60 (1.72)	95 (0.78)	
No	3420 (98.28)	12,011 (99.22)	
History of FOBT within 5 years [*n* (%)]			< 0.001
Yes	79 (2.27)	41 (0.34)	
No	3292 (94.60)	11,856 (97.93)	
Unknown	109 (3.13)	209 (1.73)	
History of colonoscopy within 5 years [*n* (%)]			< 0.001
Yes	145 (4.17)	343 (2.83)	
No	3335 (95.83)	11,763 (97.17)	

Chi square (χ^2) or Student's *t*-test was used for group comparisons. ※First-degree relative (parent, sibling, child). *SD* standard deviation, *CR*C colorectal cancer, *FOBT* fecal occult blood test

Table S5 Symptoms or signs of gastrointestinal disorders or psychological issues for participants who were enrolled but did not complete all study requirements (withdrawn cohort) compared to those who completed all study requirements (eligible cohort) [*n* (%)]

**Categories**	**Eligible cohort (*n* = 3480)**	**Withdrawn cohort (*n* = 12,106)**	***P*-value**
History of chronic diarrhoea			< 0.001
Yes	106 (3.05)	206 (1.70)	
No	3374 (96.95)	11,900 (98.30)	
History of chronic constipation			< 0.001
Yes	207 (5.95)	488 (4.03)	
No	3273 (94.05)	11,618 (95.97)	
Mucous stool			< 0.001
Yes	139 (3.99)	256 (2.11)	
No	3341 (96.01)	11,850 (97.89)	
Recent blood in stool			< 0.001
Yes	84 (2.41)	149 (1.23)	
No	3396 (97.59)	11,957 (98.77)	
Recent abdominal pain			< 0.001
Yes	113 (3.25)	189 (1.56)	
No	3367 (96.75)	11,917 (98.44)	
History of appendicitis or appendectomy			0.051
Yes	91 (2.61)	248 (2.05)	
No	3389 (97.39)	11,858 (97.95)	
History of cholecystitis or cholecystectomy			0.001
Yes	55 (1.58)	110 (0.91)	
No	3425 (98.42)	11,996 (99.09)	
Traumatized by major changes			0.021
Yes	76 (2.18)	192 (1.59)	
No	3404 (97.82)	11,914 (98.41)	
Chronic stress			< 0.001
Yes	165 (4.74)	364 (3.01)	
No	3315 (95.26)	11,742 (96.99)	

Chi square (χ^2) was used for group comparisons

Table S6 Demographic and socioeconomic risk factors and symptoms or signs of gastrointestinal disorders or psychological issues for screening-positive (FIT positive and/or miR-92a positive) participants who were enrolled but did not complete all study requirements (withdrawn cohort) compared to those who completed all study requirements (eligible cohort)

**Categories**	**Eligible cohort (*n* = 1401)**	**Withdrawn cohort (*n* = 1527)**	***P*-value**
Demographic & Socioeconomic factors			
Age (year, mean ± SD)	54.5 ± 11.2	53.3 ± 12.2	0.006
Sex [*n* (%)]			0.666
Male	647 (46.18)	692 (45.32)	
Female	754 (53.82)	835 (54.68)	
Smoking [*n* (%)]			0.210
Previous smoker	182 (12.99)	173 (11.33)	
Non-smoker	1216 (86.80)	1353 (88.61)	
Unknown	3 (0.21)	1 (0.06)	
Diabetes history [*n* (%)]			0.227
Yes	21 (1.50)	13 (0.85)	
No	1380 (98.50)	1388 (99.15)	
Cancer history other than CRC [*n* (%)]			1.000
Yes	6 (0.43)	6 (0.39)	
No	1395 (99.57)	1521 (99.61)	
Family history of CRC※ [*n* (%)]			0.016
Yes	22 (1.57)	9 (0.59)	
No	1379 (98.43)	1518 (99.41)	
History of FOBT within 5 years [*n* (%)]			< 0.001
Yes	42 (3.00)	14 (0.92)	
No	1313 (93.72)	1489 (97.51)	
Unknown	46 (3.28)	24 (1.57)	
History of colonoscopy within 5 years [*n* (%)]			0.523
Yes	44 (3.14)	38 (2.49)	
No	1357 (96.86)	1489 (97.51)	
Symptoms or Signs			
History of chronic diarrhoea [*n* (%)]			0.033
Yes	39 (2.78)	24 (1.57)	
No	1362 (97.22)	1503 (98.43)	
History of chronic constipation [*n* (%)]			0.002
Yes	90 (6.42)	58 (3.80)	

Categories	Eligible cohort (*n* = 1401)	Withdrawn cohort (*n* = 1527)	*P*-value
No	1311 (93.58)	1469 (96.20)	
Mucous stool [*n* (%)]			0.001
Yes	56 (4.00)	29 (1.90)	
No	1345 (96.00)	1498 (98.10)	
Recent blood in stool [*n* (%)]			0.001
Yes	45 (3.21)	20 (1.31)	
No	1356 (96.79)	1507 (98.69)	
Recent abdominal pain [*n* (%)]			0.002
Yes	45 (3.21)	22 (1.44)	
No	1356 (96.79)	1505 (98.56)	
History of appendicitis or appendectomy [*n* (%)]			0.184
Yes	40 (2.86)	31 (2.03)	
No	1361 (97.14)	1496 (97.97)	
History of cholecystitis or cholecystectomy [*n* (%)]			0.004
Yes	24 (1.71)	8 (0.52)	
No	1377 (98.29)	1519 (99.48)	
Traumatized by major changes [*n* (%)]			0.196
Yes	30 (2.14)	22 (1.44)	
No	1371 (97.86)	1505 (98.56)	
Chronic stress [*n* (%)]			0.344
Yes	56 (4.00)	50 (3.27)	
No	1345 (96.00)	1477 (96.73)	

Chi square (χ^2) or Student's *t*-test was used for group comparisons. ※First-degree relative (parent, sibling, child). *SD* standard deviation, *CRC* colorectal cancer, *FOBT* Fecal occult blood test

Table S7 Demographic and socioeconomic risk factors and symptoms or signs of gastrointestinal disorders or psychological issues for screening double-negative (FIT negative and miR-92a negative) participants who were enrolled but did not complete all study requirements (withdrawn cohort) compared to those who completed all study requirements (eligible cohort)

**Categories**	**Eligible cohort (*n* = 2079)**	**Withdrawn cohort (*n* = 10,579)**	***P*-value**
Demographic & Socioeconomic factors			
Age (year, mean ± SD)	53.5 ±10.5	50.8 ± 11.9	< 0.001
Sex [*n* (%)]			< 0.001
Male	814 (39.15)	4919 (46.50)	
Female	1265 (60.85)	5660 (53.50)	
Smoking [*n* (%)]			0.016
Previous smoker	211 (10.15)	1078 (10.19)	
Non-smoker	1857 (89.32)	9481 (89.62)	
Unknown	11 (0.53)	20 (0.19)	
Diabetes history [*n* (%)]			0.218
Yes	25 (1.20)	94 (0.89)	
No	2054 (98.80)	10,485 (99.11)	
Cancer history other than CRC [*n* (%)]			0.142
Yes	3 (0.14)	40 (0.38)	
No	2076 (99.86)	10,539 (99.62)	
Family history of CRC[※] [*n* (%)]			< 0.001
Yes	38 (1.83)	86 (0.81)	
No	2041 (98.17)	10,493 (99.19)	
History of FOBT within 5 years [*n* (%)]			< 0.001
Yes	37 (1.78)	27 (0.26)	
No	1979 (95.19)	10,367 (98.00)	
Unknown	63 (3.03)	185 (1.74)	
History of colonoscopy within 5 years [*n* (%)]			< 0.001
Yes	101 (4.86)	306 (2.89)	
No	1978 (95.14)	10,273 (96.92)	
Symptoms or Signs			
History of chronic diarrhoea [*n* (%)]			< 0.001
Yes	67 (3.22)	182 (1.72)	
No	2012 (96.78)	10,397 (98.28)	
History of chronic constipation [*n* (%)]			0.002
Yes	117 (5.63)	430 (4.06)	

Categories	Eligible cohort (n = 2079)	Withdrawn cohort (n = 10,579)	P-value
No	1962 (94.37)	10,149 (95.94)	
Mucous stool [n (%)]			< 0.001
Yes	83 (3.99)	227 (2.15)	
No	1996 (96.01)	10,352 (97.85)	
Recent blood in stool [n (%)]			0.022
Yes	39 (1.88)	129 (1.22)	
No	2040 (98.12)	10,450 (98.78)	
Recent abdominal pain [n (%)]			< 0.001
Yes	68 (3.27)	167 (1.58)	
No	2011 (96.73)	10,412 (98.42)	
History of appendicitis or appendectomy [n (%)]			0.280
Yes	51 (2.45)	217 (2.05)	
No	2028 (97.55)	10,362 (97.95)	
History of cholecystitis or cholecystectomy [n (%)]			0.042
Yes	31 (1.49)	102 (0.96)	
No	2048 (98.51)	10,477 (99.04)	
Traumatized by major changes [n (%)]			0.063
Yes	46 (2.21)	170 (1.61)	
No	2033 (97.79)	10,409 (98.39)	
Chronic stress [n (%)]			< 0.001
Yes	109 (5.24)	314 (2.97)	
No	1970 (94.78)	10,265 (97.03)	

Chi square (χ^2) or Student's t-test was used for group comparisons. ※First-degree relative (parent, sibling, child). *SD* standard deviation, *CRC* colorectal cancer, *FOBT* fecal occult blood test, *FIT* immunochemical fecal occult blood test

Table S8 Logistic regression model to assess the impact of dropout on the FIT, miR-92a and FIT + miR-92a sensitivity and specificity [% (95% CI)]

**Performance characteristic**	**Eligible cohort (*n* = 3480)**	**Expanded cohort (*n* =15,586)**
FIT		
AN sensitivity	54.3 (47.3 − 61.2)	52.7 (48.4 − 57.1)
AN specificity	80.7 (79.3 − 82.0)	90.7 (90.2 − 91.2)
PPV for AN	14.6 (12.3 − 16.9)	16.6 (14.2 − 19.1)
NPV for AN	96.7 (96.1 − 97.3)	98.2 (98.1 − 98.3)
miR-92a		
AN sensitivity	70.9 (64.5 − 77.2)	66.3 (62.2 − 70.8)
AN specificity	77.0 (75.6 − 78.5)	91.9 (91.5 − 92.4)
PPV for AN	15.8 (13.5 − 17.9)	22.3 (20.1 − 24.5)
NPV for AN	97.8 (97.2 − 98.3)	98.7 (98.6 − 98.8)
FIT + miR-92a		
AN sensitivity	85.4 (80.5 − 90.3)	81.7 (79.3 − 84.0)
AN specificity	62.5 (60.8 − 64.1)	93.8 (93.2 − 94.4)
PPV for AN	12.1 (10.5 − 13.8)	16.6 (15.0 − 18.3)
NPV for AN	98.6 (98.1 − 99.1)	99.7 (99.6 − 99.8)

We constructed XGBoost models for screen-positive and screen-negative individuals separately to account for the effects of factors leading to dropout (i.e., between subjects who dropped out and those who were eligible) (**Fig. S2**). A multivariate logistic regression was used to model missingness as a function of demographics and socioeconomic factors such as age, sex, and family history of CRC and different high-risk symptoms or signs such as chronic diarrhoea, chronic constipation, mucous stool, recent blood in stool, recent abdominal pain, history of cholecystitis or cholecystectomy and chronic stress. Participants who were eligible for this analysis were those who were enrolled (*n* = 15,586) less those who withdrew consent or failed to meet eligibility criteria. In total, the expanded cohort included 15,586 participants. Using the multivariate logistic regression model, the probability of having complete data was estimated for all combinations of the above variables. Variables were selected using backward exclusion with a significance threshold of $P < 0.10$. Multicollinearity was assessed by variance inflation factor (VIF), and the VIF for all variables was less than 5.0, indicating no substantial covariance. Sensitivity and specificity were then calculated using inverse probability weighting of inclusion based on the predicted probabilities from the logistic model. Performance on the eligible cohort (*n* = 3480) was compared to the expanded cohort (*n* = 15,586). Confidence intervals for the eligible cohort leveraged the Clopper-Pearson exact test. Confidence intervals for the expanded cohort were calculated as 2.5th and 97.5th percentiles of 1000 bootstrap estimates.

FIT immunochemical fecal occult blood test, *FIT + miR-92a* miR-92a in combination with immunochemical fecal occult blood test, *AN* advanced neoplasia, *PPV* positive predictive value, *NPV* negative predictive value, *XGBoost* eXtreme Gradient Boosting, *CRC* colorectal cancer

Table S9 Positive rate by miR-92a test between different groups

**Characteristic**	**Total number**	**Screening-positive participants**	**Positive rate (%)**	***P*-value**
Sex				0.079
Male	7072	681	9.6	
Female	8514	894	10.5	
Age (year)				< 0.001
30 – 35	1797	138	7.7	
36 – 40	1677	133	7.9	
41 – 45	1559	143	9.2	
46 – 50	1989	186	9.4	
51 – 55	2055	216	10.5	
56 – 60	2231	251	11.3	
61 – 65	2085	240	11.5	
66 – 70	1601	201	12.6	
71 – 75	592	67	11.3	
Cigarette smoking				0.082
Yes	1644	190	11.6	
No	13,907	1383	9.9	
Unknown	35	2	5.7	
Cancer history other than CRC				0.377
Yes	55	8	14.5	
No	15,531	1567	10.1	
Family history of CRC[a]				0.201
Yes	155	21	13.5	
No	15,431	1554	10.1	
History of FOBT within 5 years				< 0.001
Yes (positive result)	97	36	37.1	
Yes (negative result)	23	8	34.8	
No	15,148	1492	9.8	
Unknown	318	39	12.3	
History of colonoscopy within 5 years				0.903
Yes	488	48	9.8	
No	15,098	1527	10.1	
Symptoms or signs of gastrointestinal disorders				0.027
Yes	1774	206	11.6	
No	13,812	1369	9.9	

**Characteristic**	**Total number**	**Screening-positive participants**	**Positive rate (%)**	***P*-value**
Symptoms or signs of psychological issues				0.450
Yes	377	43	11.4	
No	15,209	1532	10.1	

[a]First-degree relative (parent, sibling, child).

CRC colorectal cancer, *FOBT* fecal occult blood test

Table S10 Compliance rates among high-risk population between different groups

Characteristic	High-risk participants	High-risk participants undergoing colonoscopy	Compliance rate (%)	*P* -value
Sex				0.666
Male	1339	647	48.3	
Female	1589	754	47.5	
Age (years)				0.002
30 – 35	264	103	39.0	
36 – 40	257	99	38.5	
41 – 45	247	123	49.8	
46 – 50	319	159	49.8	
51 – 55	376	193	51.3	
56 – 60	429	218	50.8	
61 – 65	481	245	50.9	
66 – 70	408	198	48.5	
71 – 75	147	63	42.9	
Cigarette smoking				0.105
Yes	355	182	51.3	
No	2569	1216	47.3	
Unknown	4	3	75.0	
Cancer history other than CRC				0.998
Yes	12	6	50.0	
No	2916	1395	47.8	
Family history of CRC[a]				0.010
Yes	31	22	71.0	
No	2897	1379	47.6	
History of FOBT within 5 years				< 0.001
Yes (positive result)	45	34	75.6	
Yes (negative result)	11	8	72.7	
No	2802	1313	46.9	
Unknown	70	46	65.7	
History of colonoscopy within 5 years				0.282
Yes	81	44	54.3	
No	2843	1354	47.6	
Symptoms or signs of gastrointestinal disorders				< 0.001
Yes	383	236	61.6	

**Characteristic**	**High-risk participants**	**High-risk participants undergoing colonoscopy**	**Compliance rate (%)**	***P* -value**
No	2545	1165	45.8	
Symptoms or signs of psychological issues				0.105
Yes	158	86	54.4	
No	2770	1315	47.5	

High-risk participants: subjects who screened positive for FIT or miR-92a.

CRC colorectal cancer, *FOBT* fecal occult blood test, *FIT* immunochemical fecal occult blood test

Table S11 FIT, miR-92a and FIT + miR92a screening for AN

**Performance characteristic**	**AN (*n* =199)**	**Non-AN (*n* =3281)**	**Total number (*n* = 3480)**
FIT			
Positive	108	634	742
Negative	91	2647	2738
miR-92a			
Positive	141	754	895
Negative	58	2527	2585
FIT + miR-92a			
Positive	170	1231	1401
Negative	29	2050	2079

Application of FIT, miR-92a, and FIT + miR-92a to screen for AN, including the number of correct and incorrect predictions.

AN advanced neoplasia, *FIT* immunochemical fecal occult blood test, *FIT + miR-92a* immunochemical fecal occult blood test combined with miR-92a test.

Table S12 Sensitivity and specificity of the miR-92a and FIT + miR-92a test by age [% (95% CI)]

Age (year)	Sensitivity			Specificity		*P*-value
	**CRC (*n* = 19)**	**AA (*n* = 180)**	**AN (*n* =199)**	**Polyps (*n*= 1851)**	**NEG (*n* = 1430)**	
FIT						< 0.001
30 – 35	–	50.0 (23.4 − 67.8)	50.0 (23.4 − 67.8)	71.6 (65.2 − 77.3)	76.6 (70.1 − 81.2)	
36 – 40	100.0 (2.5 − 100.0)	50.0 (23.4 − 67.8)	66.7 (61.9 − 73.2)	74.8 (68.8 − 79.6)	83.8 (78.9 − 88.0)	
41 – 45	100.0 (2.5 − 100.0)	71.4 (65.6 − 78.0)	75.0 (66.7 − 81.2)	78.8 (73.2 − 83.4)	84.4 (79.4 − 89.5)	
46 – 50	–	53.8 (45.8 − 57.0)	53.8 (45.8 − 57.0)	83.6 (77.8 − 88.8)	84.7 (79.0 − 89.3)	
51 – 55	100.0 (3.5 − 100.0)	58.3 (47.5 − 58.3)	61.5 (55.8 − 67.4)	81.6 (75.3 − 86.5)	87.9 (82.0 − 92.5)	
56 – 60	50.0 (23.4 − 67.8)	52.9 (46.0 − 57.4)	52.8 (45.7 − 57.4)	78.9 (73.4 − 84.1)	88.8 (83.0 − 92.3)	
61 – 65	75.0 (62.1 − 87.1)	56.3 (51.2 − 63.4)	57.7 (51.0 − 65.2)	78.7 (72.9 − 83.0)	82.2 (78.4 − 87.0)	
66 – 70	85.7 (67.6 − 90.0)	35.1 (30.4 − 40.9)	43.2 (37.8 − 47.7)	73.1 (68.4 − 78.5)	83.1 (78.2 − 88.9)	
71 – 75	100.0 (3.5 − 100.0)	45.5 (41.0 − 48.7)	53.8 (48.7 − 57.9)	64.7 (58.7 − 69.0)	76.5 (71.4 − 79.3)	
miR-92a						< 0.001
30 – 35	–	75.0 (58.8 − 81.2)	75.0 (58.8 − 81.2)	74.1 (68.7 − 80.0)	79.3 (74.3 − 84.3)	
36 – 40	100.0 (2.5 − 100.0)	50.0 (23.4 − 67.8)	66.7 (47.8 − 71.2)	62.6 (57.2 − 68.9)	89.2 (83.3 − 94.7)	
41 – 45	100.0 (2.5 − 100.0)	71.4 (64.5 − 77.6)	75.0 (58.8 − 81.2)	70.5 (65.2 − 77.1)	82.5 (78.0 − 87.3)	
46 – 50	–	61.5 (55.8 − 66.9)	61.5 (55.8 − 66.9)	71.8 (66.2 − 77.0)	85.7 (80.7 − 90.3)	
51 – 55	100.0 (3.5 − 100.0)	62.5 (56.7 − 66.7)	65.4 (62.3 − 68.9)	67.1 (63.2 − 72.1)	89.5 (84.2 − 94.0)	
56 – 60	100.0 (3.5 − 100.0)	67.6 (62.3 − 70.8)	69.4 (66.6 − 93.5)	70.1 (66.6 − 77.0)	92.4 (88.4 − 96.5)	
61 – 65	75.0 (62.1 − 87.1)	70.8 (65.4 − 75.1)	71.2 (66.6 − 74.3)	69.8 (64.3 − 72.9)	91.4 (86.8 − 94.9)	
66 – 70	100.0 (52.3 − 100.0)	70.3 (64.8 − 76.1)	75.0 (70.0 − 78.8)	66.1 (62.2 − 71.0)	88.5 (84.0 − 92.5)	
71 – 75	100.0 (3.5 − 100.0)	72.7 (66.6 − 78.7)	76.9 (72.3 − 79.9)	61.8 (57.2 − 66.3)	91.2 (87.4 − 95.0)	

Age (year)	Sensitivity			Specificity		*P*-value
	**CRC (*n* = 19)**	**AA (*n* = 180)**	**AN (*n* =199)**	**Polyps (*n*= 1851)**	**NEG (*n* = 1430)**	
FIT + miR-92a						0.0001
30 – 35	–	75.0 (68.8 − 81.0)	75.0 (68.8 − 81.0)	50.6 (45.7 − 55.8)	58.6 (53.2 − 61.0)	
36 – 40	100.0 (2.5 − 100.0)	50.0 (23.4 − 67.8)	66.7 (61.2 − 74.8)	48.7 (44.4 − 53.0)	74.3 (69.4 − 78.9)	
41 – 45	100.0 (2.5 − 100.0)	85.7 (81.8 − 93.4)	87.5 (82.1 − 94.3)	55.8 (51.1 − 60.0)	70.6 (65.4 − 75.7)	
46 – 50	–	84.6 (80.0 − 88.8)	84.6 (81.2 − 89.8)	61.8 (56.6 − 67.3)	71.9 (66.2 − 78.8)	
51 – 55	100.0 (3.5 − 100.0)	87.5 (83.1 − 90.3)	88.5 (84.2 − 93.1)	54.8 (48.8 − 59.6)	77.9 (72.4 − 84.1)	
56 – 60	100.0 (3.5 − 100.0)	79.4 (74.2 − 84.7)	80.6 (75.6 − 84.3)	55.0 (49.8 − 60.1)	82.1 (76.9 − 87.3)	
61 – 65	100.0 (30.5 − 100.0)	85.4 (78.8 − 89.0)	86.5 (81.3 − 89.8)	55.4 (50.0 − 61.1)	74.1 (69.9 − 78.9)	
66 – 70	100.0 (52.3 − 100.0)	81.1 (74.4 − 88.2)	84.1 (76.7 − 90.2)	46.9 (40.8 − 50.6)	76.2 (71.5 − 81.2)	
71 – 75	100.0 (3.5 − 100.0)	100.0 (67.3 − 100.0)	100.0 (74.2 − 100.0)	41.2 (37.7 − 46.5)	70.6 (66.2 − 74.0)	

Chi Square (χ^2) goodness of fit (GOF) was calculated for all subgroups within the age category.

FIT immunochemical fecal occult blood test, *FIT+miR-92a* miR-92a in combination with immunochemical fecal occult blood test, *CRC* colorectal cancer, *AA* advanced adenomas, *AN* advanced neoplasia, *NEG* other negative findings

Table S13 Sensitivity and specificity by demographic and socioeconomic risk factors of CRC [% (95% CI)]

Categories	Sensitivity			Specificity	
	**CRC (*n* = 19)**	**AA (*n* = 180)**	**AN (*n* = 199)**	**Polyps (*n* = 1851)**	**NEG (*n* = 1430)**
FIT					
Sex					
Female	80.0 (67.4 − 91.1)	41.3 (32.2 − 50.8)	43.8 (58.3 − 49.9)	78.9 (72.3 − 83.4)	85.9 (80.8 − 89.8)
Male	85.7 (81.0 − 91.2)	58.1 (43.7 − 66.7)	61.3 (55.4 − 68.5)	77.1 (71.8 − 82.0)	80.3 (75.7 − 84.7)
Smoking					
Yes	85.7 (81.4 − 90.0)	48.1 (40.0 − 56.8)	55.9 (50.0 − 63.2)	76.6 (72.2 − 80.9)	82.6 (78.4 − 86.0)
No	83.3 (77.6 − 85.9)	51.6 (43.2 − 58.7)	53.9 (47.6 − 60.1)	78.2 (72.8 − 84.0)	84.3 (79.9 − 89.6)
History of FOBT within 5 years					
Yes	100.0 (2.5 − 100.0)	70.0 (61.2 − 76.9)	72.7 (64.8 − 79.4)	75.4 (70.8 − 79.9)	90.9 (85.2 − 93.8)
No	88.2 (81.2 − 95.5)	51.5 (43.9 − 60.8)	54.9 (48.5 − 60.0)	80.6 (75.4 − 84.3)	84.1 (80.0 − 88.9)
History of colonoscopy within 5 years					
Yes	–	33.3 (19.8 − 41.2)	33.3 (19.8 − 41.2)	81.9 (77.7 − 87.2)	87.1 (82.6 − 93.4)
No	84.2 (78.9 − 89.0)	51.4 (47.0 − 56.8)	54.6 (46.9 − 60.3)	77.9 (72.3 − 83.0)	84.0 (81.2 − 87.9)
Family history of CRC※					
Yes	100.0 (2.5 − 100.0)	40.0 (33.3 − 48.9)	50.0 (46.8 − 61.1)	78.1 (73.4 − 84.0)	86.4 (81.8 − 90.3)
No	83.3 (78.6 − 88.5)	51.4 (46.8 − 68.7)	54.4 (47.7 − 60.2)	78.0 (72.9 − 82.1)	83.6 (78.4 − 88.5)
Overall	84.2 (79.8 − 89.9)	51.1 (45.4 − 56.8)	54.3 (47.3 − 59.7)	78.0 (74.3 − 83.0)	84.1 (81.3 − 88.9)
miR-92a					
Sex					
Female	80.0 (67.8 − 91.1)	68.0 (61.1 − 73.4)	68.8 (61.5 − 74.3)	66.8 (63.2 − 70.7)	87.8 (84.3 − 92.2)
Male	100.0 (70.3 − 100.0)	68.6 (62.3 − 73.1)	72.3 (66.8 − 78.1)	70.8 (64.5 − 75.0)	87.7 (82.6 − 93.1)

Categories	Sensitivity			Specificity	
	**CRC (*n* = 19)**	**AA (*n* = 180)**	**AN (*n* = 199)**	**Polyps (*n* = 1851)**	**NEG (*n* = 1430)**
Smoking					
Yes	100.0 (60.3 − 100.0)	74.1 (68.7 − 79.8)	79.4 (74.3 − 84.9)	66.8 (63.2 − 70.7)	88.7 (83.4 − 94.0)
No	91.7 (86.7 − 96.0)	67.3 (62.3 − 74.0)	69.1 (62.0 − 74.5)	69.0 (62.1 − 73.3)	87.7 (83.0 − 92.3)
History of FOBT within 5 years					
Yes	100.0 (2.5 − 100.0)	50.0 (43.8 − 58.3)	54.5 (43.3 − 60.8)	63.2 (58.7 − 68.9)	72.7 (66.8 − 77.9)
No	94.1 (87.8 − 99.3)	69.1 (64.3 − 74.8)	71.4 (66.3 − 76.9)	68.9 (62.3 − 74.5)	87.9 (81.7 − 92.2)
History of colonoscopy within 5 years					
Yes	−	100.0 (3.5 − 100.0)	100.0 (3.5 − 100.0)	76.4 (71.4 − 81.0)	88.6 (83.4 − 94.0)
No	94.7 (89.0 − 99.7)	67.8 (62.2 − 73.1)	70.4 (64.8 − 78.1)	68.4 (62.2 − 73.1)	87.7 (82.3 − 92.1)
Family history of CRC※					
Yes	100.0 (2.5 − 100.0)	100.0 (30.5 − 100.0)	100.0 (30.5 − 100.0)	78.1 (72.9 − 83.0)	90.9 (84.8 − 94.9)
No	94.4 (89.4 − 98.9)	67.4 (62.8 − 73.9)	69.9 (62.7 − 73.3)	68.2 (62.0 − 73.1)	87.2 (82.1 − 94.3)
Overall	94.7 (88.7 − 99.2)	68.3 (61.3 − 73.6)	70.9 (61.8 − 82.5)	68.7 (63.4 − 75.1)	87.8 (83.3 − 92.4)
FIT + miR-92a					
Sex					
Female	100.0 (40.5 − 100.0)	77.3 (72.3 − 83.4)	78.8 (73.2 − 84.1)	52.9 (47.8 − 56.9)	75.7 (70.0 − 80.2)
Male	100.0 (70.3 − 100.0)	88.6 (83.1 − 94.8)	89.9 (83.8 − 94.0)	54.8 (49.8 − 57.9)	69.4 (64.1 − 74.4)
Smoking					
Yes	100.0 (60.3 − 100.0)	88.9 (83.2 − 95.1)	91.2 (84.0 − 96.7)	51.2 (47.3 − 56.5)	72.2 (66.7 − 77.3)
No	100.0 (78.5 − 100.0)	83.0 (77.5 − 89.4)	84.2 (78.7 − 89.0)	54.2 (49.0 − 58.9)	73.8 (68.7 − 77.9)
History of FOBT within 5 years					
Yes	100.0 (2.5 − 100.0)	70.0 (58.8 − 78.5)	72.7 (67.7 − 77.9)	47.4 (42.1 − 53.0)	63.6 (58.8 − 68.7)

Categories	Sensitivity			Specificity	
	**CRC (*n* = 19)**	**AA (*n* = 180)**	**AN (*n* = 199)**	**Polyps (*n* = 1851)**	**NEG (*n* = 1430)**
No	100.0 (78.5 − 100.0)	84.8 (78.6 − 89.5)	86.3 (81.2 − 90.0)	54.1 (50.0 − 58.2)	73.8 (68.8 − 77.5)
History of colonoscopy within 5 years					
Yes	−	100.0 (3.5 − 100.0)	100.0 (3.5 − 100.0)	63.9 (59.2 − 67.6)	78.6 (72.2 − 82.1)
No	100.0 (81.0 − 100.0)	78.7 (72.3 − 84.1)	85.2 (80.3 − 91.7)	53.4 (48.7 − 56.9)	73.5 (70.0 − 78.2)
Family history of CRC※					
Yes	100.0 (2.5 − 100.0)	100.0 (30.5 − 100.0)	100.0 (40.5 − 100.0)	62.5 (57.8 − 66.9)	93.8 (88.1 − 97.6)
No	100.0 (81.0 − 100.0)	83.4 (77.7 − 90.2)	85.0 (77.6 − 89.9)	53.7 (48.8 − 56.9)	81.8 (75.6 − 86.8)
Overall	100.0 (84.3 − 100.0)	83.9 (77.9 − 91.7)	85.4 (78.6 − 90.4)	53.8 (49.2 − 57.9)	73.7 (69.4 − 78.3)

※First-degree relative (parent, sibling, child).

FIT immunochemical fecal occult blood test, *FIT+miR-92a* miR-92a in combination with immunochemical fecal occult blood test, *CRC* colorectal cancer, *AA* advanced adenomas, *AN* advanced neoplasia, *NEG* other negative findings

Table S14 Sensitivity and specificity by different symptoms or signs of gastrointestinal disorders or psychological issues for participants [% (95% CI)]

Categories	Sensitivity			Specificity	
	**CRC (*n* = 19)**	**AA (*n* = 180)**	**AN (*n* = 199)**	**Polyps (*n* = 1851)**	**NEG (*n* = 1430)**
FIT					
History of chronic diarrhoea					
Yes	100.0 (2.5 − 100.0)	40.0 (5.3 − 85.3)	50.0 (11.8 − 88.1)	85.7 (74.6 − 93.3)	86.5 (71.2 − 95.5)
No	83.3 (58.6 − 98.4)	51.4 (43.8 − 59.0)	54.4 (47.1 − 661.6)	77.7 (75.7 − 79.6)	84.1 (82.0 − 83.9)
History of chronic constipation					
Yes	66.7 (9.4 − 99.2)	40.0 (5.3 − 85.3)	50.0 (15.7 − 84.3)	77.8 (68.8 − 85.2)	81.3 (71.8 − 88.7)
No	87.5 (61.7 − 98.4)	51.4 (43.8 − 59.0)	54.5 (47.1 − 61.7)	78.0 (76.0 − 80.0)	84.3 (82.3 − 86.2)
Mucous stool					
Yes	100.0 (2.5 − 100.0)	66.7 (22.3 − 95.7)	71.4 (29.0 − 96.3)	84.5 (74.0 − 92.0)	83.6 (71.9 − 91.8)
No	83.3 (58.6 − 96.4)	50.6 (42.9 − 58.2)	53.6 (46.3 − 60.9)	77.8 (75.7 − 79.7)	84.1 (82.1 − 86.0)
Recent blood in stool					
Yes	50.0 (1.26 − 98.7)	50.0 (11.8 − 88.2)	50.0 (15.7 − 84.3)	77.4 (63.8 − 87.7)	69.6 (47.1 − 86.8)
No	88.2 (63.6 − 98.5)	51.1 (43.5 − 58.8)	54.5 (47.1 − 61.7)	78.0 (76.0 − 79.9)	84.4 (82.4 − 86.2)
Recent abdominal pain					
Yes	50.0 (1.3 − 98.7)	40.0 (5.3 − 85.3)	42.9 (9.9 − 81.6)	81.4 (69.1 − 90.3)	83.0 (69.2 − 92.4)
No	88.2 (63.6 − 98.5)	51.4 (43.8 − 59.0)	54.7 (47.4 − 61.9)	77.9 (75.9 − 79.8)	84.2 (82.1 − 86.1)
History of appendicitis or appendectomy					
Yes	−	66.7 (29.9 − 92.5)	66.7 (29.9 − 92.5)	70.5 (54.8 − 83.2)	86.8 (71.9 − 95.6)
No	84.2 (60.4 − 96.6)	50.3 (42.6 − 58.0)	53.7 (46.3 − 60.9)	78.2 (76.2 − 80.1)	84.1 (82.0 − 85.9)
History of cholecystitis or cholecystectomy					
Yes	−	−	−	81.5 (61.9 − 93.7)	76.0 (54.9 − 90.6)
No	84.2 (60.4 − 96.6)	52.0 (44.4 − 59.5)	55.1 (47.9 − 62.2)	78.0 (75.9 − 79.8)	84.3 (82.3 − 86.1)

Categories	Sensitivity			Specificity	
	**CRC (*n* = 19)**	**AA (*n* = 180)**	**AN (*n* = 199)**	**Polyps (*n* = 1851)**	**NEG (*n* = 1430)**
Traumatized by major changes					
Yes	100.0 (2.5 − 100.0)	40.0 (5.3 − 85.3)	50.0 (11.8 − 88.2)	78.0 (62.4 − 89.4)	89.7 (75.4 − 98.2)
No	83.3 (68.9 − 95.6)	51.4 (43.2 − 57.4)	54.4 (56.4 − 60.0)	78.0 (76.0 − 80.1)	84.0 (81.9 − 85.8)
Chronic stress					
Yes	100.0 (2.5 − 100.0)	50.0 (42.2 − 57.4)	57.1 (47.9 − 62.1)	82.9 (79.7 − 83.3)	87.8 (84.5 − 88.1)
No	83.3 (67.7 − 98.9)	51.1 (43.3 − 58.8)	54.2 (46.2 − 58.7)	79.6 (78.7 − 81.7)	83.9 (82.1 − 85.9)
miR-92a					
History of chronic diarrhoea					
Yes	–	80.0 (33.9 − 97.1)	66.7 (60.0 − 75.1)	68.3 (61.1 − 71.0)	91.9 (87.5 − 93.0)
No	100.0 (81.5 − 100.0)	68.0 (53.3 − 77.9)	71.0 (63.1 − 78.8)	68.7 (61.8 − 72.1)	87.7 (84.0 − 89.3)
History of chronic constipation					
Yes	100.0 (7.5 − 100.0)	60.0 (45.7 − 67.3)	75.0 (66.1 − 80.0)	63.9 (59.8 − 67.9)	82.4 (79.0 − 84.9)
No	93.8 (78.8 − 97.6)	68.6 (56.7 − 75.2)	70.7 (61.6 − 78.9)	69.0 (66.9 − 72.2)	88.1 (86.2 − 90.8)
Mucous stool					
Yes	100.0 (2.5 − 100.0)	100.0 (23.4 − 100.0)	100.0 (37.7 − 100.0)	67.6 (59.8 − 70.2)	88.5 (85.7 − 90.8)
No	94.4 (78.5 − 98.9)	67.2 (57.2 − 73.3)	69.8 (63.3 − 75.3)	68.8 (66.1 − 71.2)	87.7 (84.8 − 89.9)
Recent blood in stool					
Yes	100.0 (3.5 − 100.0)	100.0 (23.4 − 100.0)	100.0 (40.5 − 100.0)	64.2 (58.7 − 72.1)	87.0 (84.2 − 89.2)
No	94.1 (78.2 − 98.7)	67.2 (57.1 − 73.4)	69.6 (63.1 − 75.1)	68.9 (66.2 − 71.4)	87.8 (85.0 − 89.9)
Recent abdominal pain					
Yes	100.0 (2.5 − 100.0)	80.0 (67.8 − 89.0)	85.7 (70.8 − 90.2)	76.3 (70.4 − 80.1)	74.5 (69.0 − 79.9)
No	94.0 (78.8 − 98.5)	68.0 (58.9 − 77.7)	70.3 (60.9 − 77.8)	68.5 (66.1 − 70.9)	88.2 (86.0 − 90.3)
History of appendicitis or appendectomy					
Yes	–	77.8 (66.6 − 80.1)	77.8 (66.6 − 80.1)	63.6 (43.1 − 78.0)	94.7 (82.1 − 98.7)

Categories	Sensitivity			Specificity	
	**CRC (*n* = 19)**	**AA (*n* = 180)**	**AN (*n* = 199)**	**Polyps (*n* = 1851)**	**NEG (*n* = 1430)**
No	100.0 (86.3 − 100.0)	67.8 (59.9 − 76.2)	71.1 (1.2 − 78.3)	68.8 (65.5 − 70.3)	87.6 (84.9 − 90.0)
History of cholecystitis or cholecystectomy					
Yes	−	100.0 (3.5 − 100.0)	100.0 (3.5 − 100.0)	66.7 (59.0 − 73.4)	92.0 (88.1 − 94.4)
No	94.7 (90.9 − 96.5)	67.8 (56.8 − 77.1)	70.4 (66.7 − 78.9)	68.8 (62.0 − 75.1)	87.7 (86.1 − 93.2)
Traumatized by major changes					
Yes	100.0 (2.5 − 100.0)	100.0 (30.5 − 100.0)	100.0 (30.5 − 100.0)	65.9 (63.0 − 68.8)	89.7 (78.8 − 93.0)
No	94.0 (80.6 − 98.9)	67.4 (63.0 − 73.4)	69.9 (63.5 − 75.0)	68.8 (63.8 − 72.1)	87.7 (86.1 − 93.3)
Chronic stress					
Yes	100.0 (2.5 − 100.0)	100.0 (30.5 − 100.0)	100.0 (40.5 − 100.0)	67.1 (63.8 − 69.4)	90.2 (83.1 − 94.1)
No	94.0 (80.6 − 98.9)	67.2 (62.3 − 71.9)	69.8 (63.4 − 74.3)	70.4 (66.6 − 74.2)	87.6 (82.3 − 90.0)
FIT + miR-92a					
History of chronic diarrhoea					
Yes	100.0 (2.5 − 100.0)	80.0 (73.3 − 88.7)	83.3 (67.7 − 88.0)	57.1 (55.4 − 60.0)	81.1 (76.7 − 85.0)
No	100.0 (81.5 − 100.0)	84.0 (77.8 − 87.6)	89.9 (83.2 − 90.0)	53.7 (47.8 − 57.0)	73.5 (70.0 − 77.7)
History of chronic constipation					
Yes	100.0 (3.5 − 100.0)	80.0 (73.3 − 88.7)	87.5 (77.7 − 93.9)	50.0 (47.5 − 52.3)	68.1 (62.0 − 74.1)
No	100.0 (76.3 − 100.0)	84.0 (77.8 − 87.6)	85.3 (83.1 − 89.7)	54.0 (51.1 − 56.9)	74.1 (70.4 − 78.8)
Mucous stool					
Yes	100.0 (2.5 − 100.0)	100.0 (30.5 − 100.0)	100.0 (40.5 − 100.0)	54.9 (52.2 − 55.7)	72.1 (66.6 − 76.9)
No	100.0 (81.5 − 100.0)	83.3 (81.2 − 87.7)	84.9 (81.7 − 88.9)	53.8 (50.0 − 56.0)	73.8 (70.0 − 76.7)
Recent blood in stool					
Yes	100.0 (2.5 − 100.0)	100.0 (30.5 − 100.0)	100.0 (40.5 − 100.0)	49.1 (46.1 − 54.3)	56.5 (44.8 − 70.0)
No	100.0 (79.5 − 100.0)	83.3 (81.2 − 87.7)	84.8 (82.3 − 88.8)	53.9 (49.8 − 56.6)	74.0 (70.9 − 78.1)
Recent abdominal pain					

Categories	Sensitivity			Specificity	
	**CRC (*n* = 19)**	**AA (*n* = 180)**	**AN (*n* = 199)**	**Polyps (*n* = 1851)**	**NEG (*n* = 1430)**
Yes	100.0 (2.5 − 100.0)	100.0 (18.5 − 100.0)	100.0 (40.5 − 100.0)	64.4 (61.3 − 67.0)	63.8 (59.8 − 67.8)
No	100.0 (79.5 − 100.0)	83.4 (81.4 − 87.8)	84.9 (81.7 − 88.9)	53.5 (49.7 − 56.0)	74.0 (71.4 − 78.8)
History of appendicitis or appendectomy					
Yes	−	100.0 (47.3 − 100.0)	100.0 (47.3 − 100.0)	43.2 (39.8 − 48.0)	84.2 (81.3 − 88.5)
No	94.7 (87.7 − 98.8)	83.0 (81.0 − 86.7)	84.2 (81.0 − 87.6)	54.1 (53.2 − 58.1)	73.4 (70.2 − 77.4)
History of cholecystitis or cholecystectomy					
Yes	−	100.0 (3.5 − 100.0)	100.0 (3.5 − 100.0)	48.1 (44.2 − 54.0)	72.0 (68.7 − 75.5)
No	100.0 (86.2 − 100.0)	83.6 (82.4 − 87.9)	85.2 (82.3 − 88.5)	53.9 (49.9 − 57.7)	73.7 (71.1 − 76.3)
Traumatized by major changes					
Yes	100.0 (2.5 − 100.0)	100.0 (30.5 − 100.0)	100.0 (30.5 − 100.0)	53.7 (51.1 − 55.4)	82.8 (81.2 − 84.3)
No	100.0 (81.5 − 100.0)	83.4 (81.4 − 87.9)	85.0 (82.3 − 88.8)	53.8 (49.6 − 57.6)	73.5 (70.7 − 75.6)
Chronic stress					
Yes	100.0 (2.5 − 100.0)	100.0 (30.5 − 100.0)	100.0 (40.5 − 100.0)	56.6 (50.8 − 59.3)	80.5 (76.8 − 84.3)
No	100.0 (81.5 − 100.0)	83.3 (81.2 − 87.7)	84.9 (81.1 − 89.7)	55.0 (49.8 − 58.7)	73.3 (71.3 − 76.8)

FIT immunochemical fecal occult blood test, *FIT + miR-92a* miR-92a in combination with immunochemical fecal occult blood test, *NEG* other negative findings.

Table S15 Sensitivity of FIT, miR-92a and FIT + miR-92a for subcategories of colorectal cancer (CRC) (*n* = 19) and advanced adenomas (AA) (*n* = 180) [% (95%CI)]

**Categories**	**FIT**	**miR-92a**	**FIT + miR-92a**
CRC sensitivity by stage			
Stage I/II	100.0 (2.5 − 100.0)	100.0 (2.5 − 100.0)	100.0 (2.5 − 100.0)
Stage III/IV	–	100.0 (2.5 − 100.0)	100.0 (2.5 − 100.0)
Unknown	88.2 (63.6 − 98.5)	94.1 (71.3 − 99.9)	100.0 (80.5 − 100.0)
AA sensitivity by histology			
TVA, any size	46.3 (30.6 − 62.6)	58.5 (42.1 − 73.7)	80.5 (65.1 − 91.2)
TA, any size	46.5 (34.5 − 58.7)	63.4 (51.1 − 74.5)	81.7 (70.7 − 89.9)
TSA, any size	100.0 (15.8 − 100.0)	100.0 (15.8 − 100.0)	100.0 (15.8 − 100.0)
AA sensitivity by size			
< 10 mm	31.3 (11.0 − 58.7)	56.3 (29.9 − 80.2)	75.0 (47.6 − 92.7)
10 – 20 mm	49.6 (40.8 − 58.5)	73.3 (64.8 − 80.6)	84.7 (77.4 − 90.4)
> 20 mm	68.8 (50.0 − 83.9)	53.1 (37.4 − 70.9)	84.4 (67.2 − 94.7)
AA sensitivity by location			
Distal to and inclusive of the splenic flexure	45.3 (31.6 − 59.6)	67.9 (53.7 − 80.1)	83.0 (70.2 − 91.9)
Proximal to the splenic flexure	50.0 (26.6 − 75.3)	75.0 (47.6 − 92.7)	87.5 (61.7 − 98.4)
Proximal and distal multiple	54.1 (44.3 − 63.6)	67.6 (58.0 − 76.1)	83.8 (75.6 − 90.1)

FIT immunochemical fecal occult blood test, *FIT + miR-92a* miR-92a in combination with immunochemical fecal occult blood test, *TVA* tubulovillous adenoma, *TA* tubular adenoma, *TSA* traditional serrated adenomas

Table S16 Sensitivity and specificity of different screening strategies for screening AN (*n* = 3415) [% (95% CI)]

Performance characteristic	FIT	miR-92a	FIT + miR-92a
Sensitivity	53.4 (46.0 – 60.8)	71.4 (64.6 – 77.8)	86.2 (81.5 – 91.0)
Specificity	80.7 (79.3 – 82.0)	77.2 (75.7 – 78.7)	62.6 (60.9 – 64.5)

Analysis performed after excluding subjects who had FOBT with a positive result within 5 years (*n* = 3415).

FIT immunochemical fecal occult blood test, *FIT + miR-92a* immunochemical fecal occult blood test combined with miR-92a test, *FOBT* fecal occult blood test

Table S17 Positive predictive value (PPV) and negative predictive value (NPV) for FIT, miR-92a and FIT + miR-92a

Categories	Ratio	Estimate [% (95% CI)]
FIT		
PPV (CRC)	16/742	2.2 (1.8 – 2.7)
PPV (AA)	92/742	12.4 (11.3 – 13.5)
PPV (AN)	108/742	14.6 (12.2 – 17.3)
NPV (non-CRC and non-AA)	2647/2738	96.7 (95.9 – 97.3)
miR-92a		
PPV (CRC)	18/895	2.0 (1.6 – 2.5)
PPV (AA)	123/895	13.7 (12.6 – 14.9)
PPV (AN)	141/895	15.8 (13.5 – 18.3)
NPV (non-CRC and non-AA)	2527/2585	97.8 (97.1 – 98.3)
FIT + miR-92a		
PPV (CRC)	19/1401	1.4 (1.1 – 1.9)
PPV (AA)	151/1401	10.8 (9.8 – 11.9)
PPV (AN)	170/1401	12.1 (10.4 – 13.8)
NPV (non-CRC and non-AA)	2050/2079	98.6 (98.1 – 99.1)

Out of 3480 participants who underwent colonoscopy, 742 subjects tested positive for FIT, with 16 CRC, 92 AA, and 108 AN (CRC or AA) cases diagnosed. 895 subjects tested positive for miR-92a, with 18 CRC, 123 AA, and 141 AN cases diagnosed. 1401 subjects tested positive for FIT + miR-92a, with 19 CRC, 151 AA, and 170 AN cases diagnosed. Although PPV demonstrated superior results compared to other colorectal cancer screening methods, its effectiveness was also limited by the low incidence of AN in the general risk population. Among the 2738 participants with a negative FIT result, 2647 were determined to not have CRC or AA. Among the 2585 participants with a negative miR-92a result, 2527 were determined to not have CRC or AA. Among the 2079 participants with a negative FIT result, 2050 were determined to not have CRC or AA.

FIT immunochemical fecal occult blood test, *FIT + miR-92a* miR-92a in combination with immunochemical fecal occult blood test, *CRC* colorectal cancer, *AA* advanced adenomas, *AN* advanced neoplasia, *PPV* positive predictive value, *NPV* negative predictive value

Table S18 Diagnostic likelihood ratios (DLRs) for FIT, miR-92a and FIT + miR-92a [% (95% CI)]

**Categories**	**Positive DLR**	**Negative DLR**
FIT		
CRC vs. other findings (AA excluded)	4.36 (4.21 – 4.51)	0.20 (0.19 – 0.21)
AA vs. other findings (CRC excluded)	2.65 (2.56 – 2.74)	0.61 (0.59 – 0.63)
AN vs. other findings	2.81 (2.44 – 3.10)	0.57 (0.54 – 0.69)
miR-92a		
CRC vs. other findings (AA excluded)	4.12 (3.98 – 4.26)	0.07 (0.06 – 0.08)
AA vs. other findings (CRC excluded)	2.97 (2.87 – 3.07)	0.41 (0.40 – 0.42)
AN vs. other findings	3.08 (2.78 – 3.38)	0.38 (0.34 – 0.49)
FIT + miR-92a		
CRC vs. other findings (AA excluded)	2.67 (2.26 – 3.01)	–
AA vs. other findings (CRC excluded)	2.24 (2.10 – 2.67)	0.26 (0.25 – 0.27)
AN vs. other findings	2.27 (2.14 – 2.58)	0.23 (0.12 – 0.46)

95% CIs were estimated using the 2.5th and 97.5th percentiles of bootstrap distributions. The results indicate that a person with CRC is 4.12 times more likely to have a positive test result than a person without CRC and a person with CRC or AA is 3.08 times more likely to have a positive test result than a person with neither CRC nor AA by miR-92a. A person with CRC is 4.35 times more likely to have a positive test result than a person without CRC and a person with CRC or AA is 3.71 times more likely to have a positive test result than a person with neither CRC nor AA by FIT + miR-92a.

FIT immunochemical fecal occult blood test, *FIT + miR-92a* miR-92a in combination with immunochemical fecal occult blood test, *CRC* colorectal cancer, *AA* advanced adenomas, *AN* advanced neoplasia, *CI* confidence intervals

Table S19 Detection rates (DRs) of CRC, AA and AN by FIT, miR-92a and FIT + miR-92a (*n* = 3480) [% (95% CI)]

Categories	CRC DRs	AA DRs	AN DRs
FIT (*n* = 742)	2.2 (1.2 – 3.5)	12.4 (10.1 – 15.0)	14.6 (12.1 – 17.3)
miR-92a (*n* = 895)	2.0 (1.2 – 3.2)	13.7 (11.6 – 16.2)	15.8 (13.4 – 18.3)
FIT + miR-92a (*n* = 1401)	1.4 (0.8 – 2.1)	10.8 (9.2 – 12.5)	12.1 (10.5 – 14.0)

95% CIs were estimated using the 2.5th and 97.5th percentiles of bootstrap distributions. Values in the table for DR are represented by %. Out of 3480 participants who underwent colonoscopy, 742 subjects tested positive for FIT, with 16 CRC, 92 AA, and 108 AN (CRC or AA) cases diagnosed. 895 subjects tested positive for miR-92a, with 18 CRC, 123 AA, and 141 AN cases diagnosed. 1401 subjects tested positive for FIT + miR-92a, with 19 CRC, 151 AA, and 170 AN cases diagnosed.

FIT immunochemical fecal occult blood test, *FIT + miR-92a* miR-92a in combination with immunochemical fecal occult blood test, *CRC* colorectal cancer, *AA* advanced adenomas, *AN* advanced neoplasia, *CIs* confidence intervals, *DRs* detection rates

Table S20 Characteristics of advanced neoplasias [*n* (%)]

**Characteristic**	**CRC**	**AA**
Number of lesions		
Single	0 (0.00)	30 (16.67)
Multiple	2 (10.53)	150 (83.33)
NA	17 (89.47)	0 (0.0)
Location of lesions		
Ascending colon	2 (10.53)	64 (14.00)
Transverse colon	1 (5.26)	84 (18.38)
Descending colon	0 (0.00)	65 (14.22)
Sigmoid colon	7 (36.84)	128 (28.01)
Cecum	0 (0.00)	24 (5.25)
Rectum	6 (31.58)	71 (15.54)
Rectosigmoid junction	3 (15.79)	7 (1.53)
Hepatic flexure	0 (0.00)	13 (2.85)
Splenic flexure	0 (0.00)	1 (0.22)
Classification of lesion location		
Distal	17 (89.47)	53 (29.44)
Proximal	2 (10.53)	16 (8.89)
Distal + Proximal	0 (0.00)	111 (61.67)
Lesion size		
0.5 – 1.0 cm	-	16 (8.89)
1.0 – 2.0 cm	-	131 (72.78)
2.0 – 3.0 cm	-	27 (15.00)
≥ 3.0 cm	-	5 (2.78)
NA	-	1 (0.55)

Lesions of AA can involve multiple locations.

CRC colorectal cancer, *AA* advanced adenoma, *NA* not available

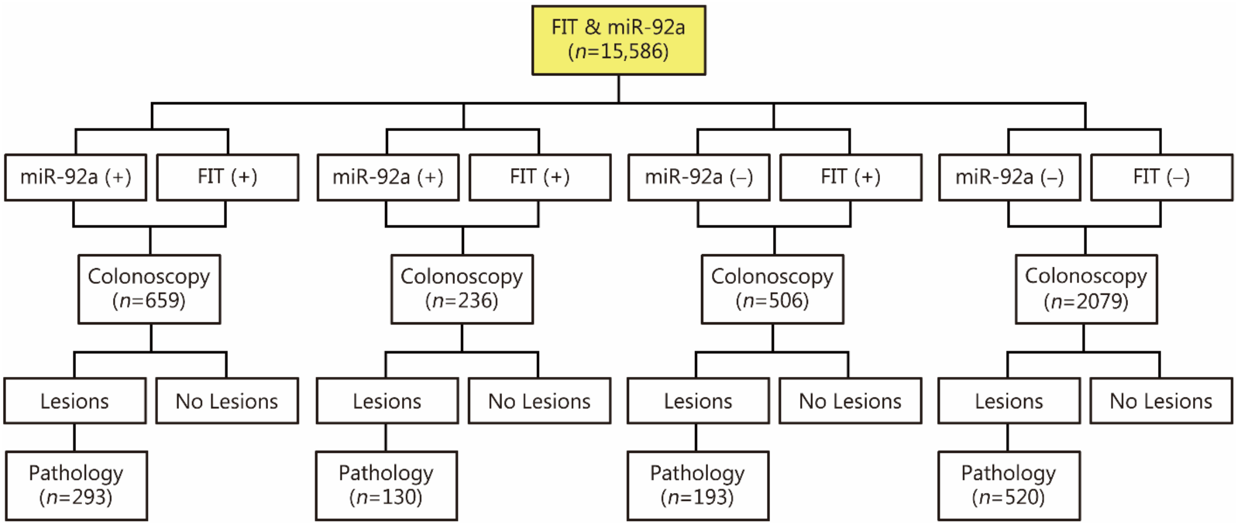

Fig. S1 Flowchart for colonoscopy and/or pathology for different screening results. In accordance with the trial design, eligible subjects were screened for FIT and/or miR-92a. Those with positive screening results and a randomly selected portion of those with negative screening results were recommended for colonoscopy and followed up until completion of the colonoscopy. FIT immunochemical fecal occult blood test

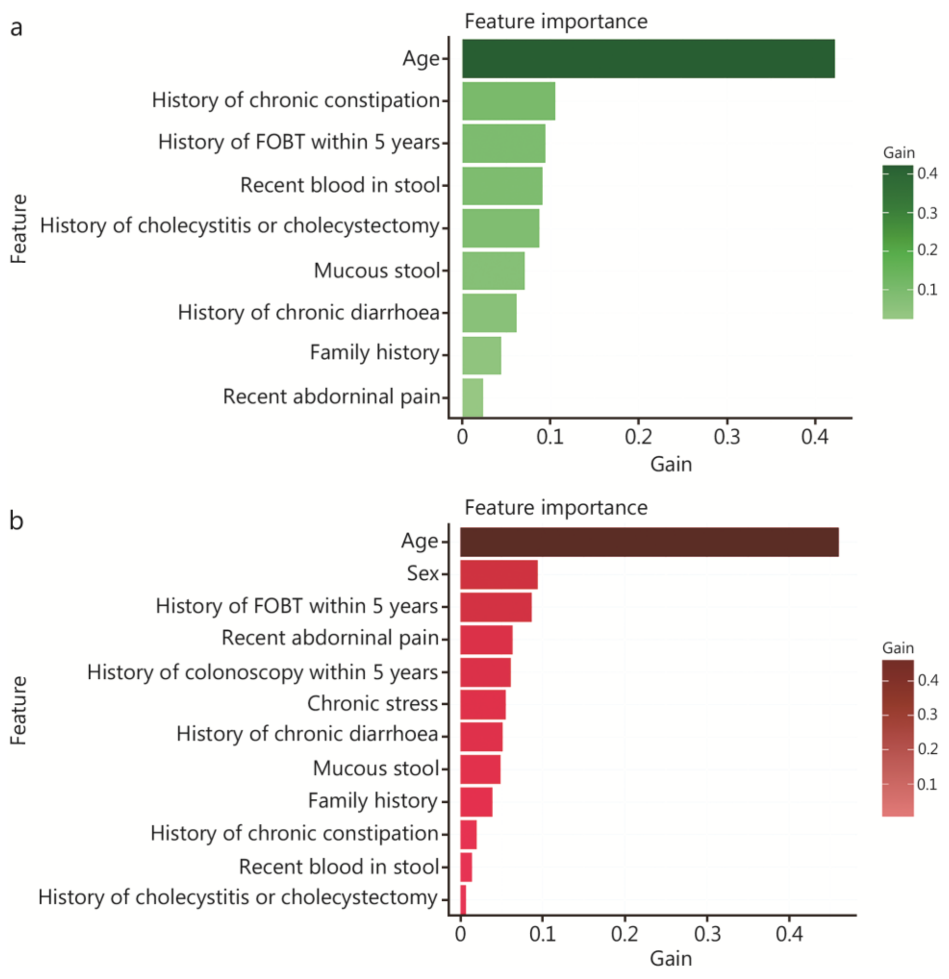

Fig. S2 XGBoost classification models illustrated the influence of various factors contributing to dropout. Model was used on participants who were withdrawn for no colonoscopy result. **a** An XGBoost classification model illustrated the impact of factors that contribute to the dropout of individuals with a positive screening result. **b** An XGBoost classification model illustrates the impact of factors that contribute to the dropout of individuals with a double-negative screening result. The XGBoost model was optimized through a grid search strategy evaluating hyperparameter combinations within predefined ranges: learning rate (eta = 0.01, 0.1), maximum tree depth (max_depth = 3, 6), boosting rounds (nrounds = 50, 100, 150), regularization (gamma = 0, 0.1), feature subsampling ratio (colsample_bytree = 0.75, 1), instance subsampling ratio (subsample = 0.75, 1), and minimum child node weight (min_child_weight = 1, 3). The optimal combination was selected via 5-fold cross-validation. To prevent overfitting, we implemented three-level regularization: 1) structural constraints through gamma-controlled split thresholds and minimum child weight requirements; 2) stochasticity via concurrent row (subsample) and column (colsample_bytree) subsampling; 3) cross-validation-based early termination without explicit stopping rounds, where the final iteration count was determined by cross-validation performance. XGBoost eXtreme gradient boosting, FOBT fecal occult blood test
